# *In Silico* Evaluation of Enzymatic Tunnels in the Biotransformation of α-Tocopherol Esters

**DOI:** 10.3389/fbioe.2021.805059

**Published:** 2022-01-21

**Authors:** Tamara Stela Mendonça Azevedo, Lavínia Kelly Barros Silva, Álvaro Silva Lima, Matheus Mendonça Pereira, Elton Franceschi, Cleide Mara Faria Soares

**Affiliations:** ^1^ Graduate Program in Industrial Biotechnology, Tiradentes University (UNIT), Aracaju, Brazil; ^2^ Institute of Technology and Research (ITP), Aracaju, Brazil; ^3^ Department of Materials and Ceramic Engineering, CICECO ‐ Aveiro Institute of Materials, University of Aveiro, Aveiro, Portugal

**Keywords:** α-tocopherol, lipase, tunnels, biotransformation, α-tocopherol esters

## Abstract

**Motivation:** α-Tocopherol is a molecule obtained primarily from plant sources that are important for the pharmaceutical and cosmetics industry. However, this component has some limitations such as sensitivity to oxygen, presence of light, and high temperatures. For this molecule to become more widely used, it is important to carry out a structural modification so that there is better stability and thus it can carry out its activities. To carry out this structural modification, some modifications are carried out, including the application of biotransformation using enzymes as biocatalysts. Thus, the application of a computational tool that helps in understanding the transport mechanisms of molecules in the tunnels present in the enzymatic structures is of fundamental importance because it promotes a computational screening facilitating bench applications.

**Objective:** The aim of this work was to perform a computational analysis of the biotransformation of α-tocopherol into tocopherol esters, observing the tunnels present in the enzymatic structures as well as the energies which correspond to the transport of molecules.

**Method:** To carry out this work, 9 lipases from different organisms were selected; their structures were analyzed by identifying the tunnels (quantity, conformation, and possibility of transport) and later the calculations of substrate transport for the biotransformation reaction in the identified tunnels were carried out. Additionally, the transport of the product obtained in the reaction through the tunnels was also carried out.

**Results:** In this work, the quantity of existing tunnels in the morphological conformational characteristics in the lipases was verified. Thus, the enzymes with fewer tunnels were RML (3 tunnels), LBC and RNL (4 tunnels), PBLL (5 tunnels), CALB (6 tunnels), HLG (7 tunnels), and LCR and LTL (8 tunnels) and followed by the enzyme LPP with the largest number of tunnels (39 tunnels). However, the enzyme that was most likely to transport substrates in terms of α-tocopherol biotransformation (in relation to the E_max_ and E_a_ energies of ligands and products) was CALB, as it obtains conformational and transport characteristics of molecules with a particularity. The most conditions of transport analysis were α-tocopherol tunnel 3 (E_max_: −4.6 kcal/mol; E_a_: 1.1 kcal/mol), vinyl acetate tunnel 1 (E_max_: −2.4 kcal/mol; E_a_: 0.1 kcal/mol), and tocopherol acetate tunnel 2 (E_max_: −3.7 kcal/mol; E_a_: 2 kcal/mol).

## Introduction

Better known as vitamin E, α-tocopherol was discovered in 1922; it is a molecule with a fat-soluble characteristic that can be extracted from different plant sources and has great importance for the body’s physiological functions acting on oxidative stress, age, inflammatory diseases, and cardiovascular diseases as well as neurodegenerative diseases (Bartolini et al., 2021). Although there are other forms of tocopherol (β, γ, and δ), which perform biological activities, only α-tocopherol has shown to be essential in the prevention of diseases and can be called a vitamin (Azzi, 2018). The most well-known characteristic of α-tocopherol is its antioxidant activity as it has the function of eliminating free radicals, donating its phenolic hydrogen present in its structure to free oxygen radicals. When applied in the topic form, α-tocopherol realizes a primordial compound function in care with pollution, UV-rays, and antiaging. However, as α-tocopherol is unstable under light, oxygen, and high temperatures, an alternative for inserting this component in formulations in a different way with better properties is to perform the structural modification (Freitas et al., 2008; Ben-Shabat et al., 2013; Abla and Banga, 2014). Further route of α-tocopherol is the structural modification to tocopherol esters since these esters have advantages over the unmodified molecule. Some esters such as acetate, succinate, ferulate, and α-tocopherol nicotinate have better stability than α-tocopherol and are more widely used in different products of the pharmaceutical industry for internal use (nutraceuticals and supplements) and topical products (products of hygiene, cosmetics, and semi-solid formulations) (Yang and McClements, 2013). The topical use, in addition to carrying out the antioxidant action, it slows down the degradation process of collagen existing in the skin, moreover to protect against ultraviolet rays, which suggests a wide use in antiaging and sun protection products (Abla and Banga, 2014). To obtain these esters, the industry uses chemical routes to obtain the final product.

The most commonly used catalysts in reactions are Lewis acid, BrÔnsted acid, or a combination of acids (ZnCl_2_, ZnCl_2_/HCl, BF_3_, AlCl_3_, and FeCl_2_/Fe/HCl). These catalysts have a low cost, but their large-scale production can generate numerous disadvantages such as wasted water, raw material, low recovery rate and catalyst use, and generating more by-products (Shi and Zhang, 2017; Zou et al., 2021).

Thus, the use of enzymatic catalysis has been a useful alternative in the reaction process with conditions of greater specificity and speed. Among the various types of enzymes that can be applied in industrial processes, lipases are biocatalysts that have different applications in industry and are considered to be quite useful for being present in industrial processes such as the food, beverage, pharmaceutical, and cosmetics industries (Sarmah et al., 2018). Therefore, obtaining a product that has a process with a more environmentally favorable route, such as the use of biocatalysts, is of great interest.

The first report of obtaining tocopherol esters by the enzymatic route was obtained by Torres et al. (2008) in which the acylation of the phenolic group of α-tocopherol was carried out through the transesterification reaction using vinyl acetate in 2-methyl-2-butanol to obtain α-tocopherol acetate using different lipases as biocatalysts. Lipase (triacylglycerol acyl hydrolase EC 3.1.1.3) has several advantages. They have the ability to carry out acetylation, transesterification, hydrolysis, and esterification reactions in order to obtain different products for a wide industrial range. They are safe for human consumption and an abundant source of essential amino acids, antioxidants, and minerals and have found application in health foods, supplements, and cosmetics. These enzymes have been extensively studied due to their different biochemical and physiological properties (Kumar et al., 2019; Kublicki et al., 2021). Since, the catalytic residues present in its triad occur in a different order in the protein sequence for lipases (Ser-His-Asp/Glu). This group of enzymes has a molecular weight ranging between 20 and 75 kDa, optimal pH for activity 4–9, and performing activity in the temperature range between 25 and 70°C (Uppenberg et al., 1994; Rios et al., 2018). The screening was performed with different lipases [lipase B from *Candida antarctica* (Novozyme 435), lipase from *Thermomyces lanuginosus* (TL IM), lipase from *Rhizomucor miehei* (RM IM), lipase from *Pseudomonas cepacia* (Lipase 1 PS-C), lipase from *Alcaligenes* sp (Lipase PLG), lipase from *Alcaligenes* sp (Lipase QLG), cholesterol esterase from *Pseudomonas* sp., cholesterol esterase from *Ophiostoma piceae* (immobilized in Dilbeads TA), lipase from porcine pancreas, lipase from *Candida rugosa*, protease from *Bacillus licheniformis*, esterases from bovine rumen microflora, and esterase from the Urania hypersaline basin]. In 2011, Chunhua et al. (2011) also synthesized enzymatically, using *Candida antarctica* lipase B (Novozym 435), to obtain tocopherol succinate, modifying the biocatalyst to improve its catalytic performance using acetic anhydride, propionic anhydride, and succinic anhydride separately, and with the best modifying agent succinic anhydride (1: 5) yielded 94.4%. Jiaojiao et al. (2021) used a nanogel-modified *Candida rugosa* lipase to obtain vitamin E succinate and obtained a yield of approximately 62% with a reaction time of 15 h.

Xin et al. (2011), on the other hand, verified the ability of lipases to catalyze through the transesterification reaction of α-tocopherol and ethyl ferulate to obtain tocopherol ferulate, and it can be observed that only *Candida antarctica* lipase B and *Candida rugosa* lipase catalyzed the transesterification reaction. A yield of 25.2% tocopherol ferulate was obtained using a 5:1 molar ratio of α-tocopherol to solvent-free ethyl ferulate in 72 h of reaction. To predict the behavior of substrates in the face of the processes that biocatalysis uses, applying molecular dynamics tools has been a useful strategy.

Computational screening is a well-established technique that is used to observe the structure of components used in the pharmaceutical industry and in the academic environment (Wolf et al., 2010). It is a strategy that assists in the first stages of the drug preparation process to generate a large database of compounds in one given receptor and then selecting the binders obtained from commercial libraries (Pinto et al., 2019; Marques et al., 2020). The tracking and analysis of the behavior of the ligand along the path of biomolecular systems in molecular dynamics represent a strategy that enhances the protein design process, to highlight important regions for the transport of ligands, that is, molecular tunnels, channels, and gates, which establish the association of the ligand with dissociation mechanisms (Surpeta et al., 2020).

Enzyme tunnels and channels are the fundamental transport pathways that allow ligands to move from the outer to the inner medium of the protein. Consequently, there are web servers that can be applied to study the transport of molecules in tunnels such as MolAxis, MOLE, ChExVis, 3V, and CAVER. Thus, the use of the Caver web server (a combination of Caver and CaverDock online) is an innovative tool to investigate the passage of the ligand through the channels present in biomolecules (Kokkonen et al., 2019; Marques et al., 2020; Singh et al., 2021). Enzymatic tunnels are characterized by having a single opening that allows the transport of substrates, products, and other molecules inside and outside the active site. These enzymatic structures are essential for a good catalytic behavior of the enzyme, as they directly affect its specificity, stability, and substrate activity. The conformation and physicochemical properties of the tunnels also function as a way of protecting the hydrophobic nucleus of the enzymes to restrict the access of solvent molecules and inhibitors (Vavra et al., 2019).

Recently, identification studies on the catalytic behavior of enzymes as fatty acids developed by our group were essential for understanding the reactions at the molecular level. However, the study of the transport of substances through enzymatic tunnels has not yet been explored. Consequently, the study of molecular dynamics in relation to biocatalysis can promote an optimization of experimental time in the bench, as these studies can predict the behavior of the biocatalyst against the substrate of interest (Barbosa et al., 2019; Brandão et al., 2020; Santana et al., 2020; Rodrigues et al., 2021). The study of tunnels to access and transport of ligands using experimental techniques *in silico* is not too investigated, not so well understood, and not yet widely used, as these analyses present a broad challenge, since most methods used to verify tunnels are based on molecular dynamics simulation practices (Stourac et al., 2019 **).**

Therefore, the objective of this work was to identify the tunnels present in the biocatalysts and their characteristics, as well as to select the tunnels with the highest probability of substrate transport (in the acetylation reaction) *in silico*, carry out the transport of products in the tunnels, identify the most viable route of obtaining, and, therefore, identify the best biocatalyst to be used in the reaction to obtain α-tocopherol esters applying molecular dynamics tools.

## Materials and Methods

To carry out this study, a screening of lipases was carried out with potential applications for the biotransformation of α-tocopherol. Altogether, 9 lipases were selected (Table 1), and with the aid of the Discovery Studio program, the amino acids that make up the active site of the right lipases were selected. For each lipase, the PDBs (Protein Data Banks) were able to verify the crystallographic structures. The SMILES (Simplified Molecular Input Line Entry System) codes available at PubChem for each molecule subjected to analysis were used (Table 2). Subsequently, the PDBs of each lipase were identified using the computer web server CaverWeb, together with the SMILES codes of the substances to analyze the possible tunnels contained in the structures as well as the possible transport of each molecule in the biocatalyst. The CaverWeb 1.0 server is available for free; it is simple to realize the analysis and allows the online execution of the steps of screening. First, the enzyme specifications were carried out followed by specifications; after which the calculation of tunnels according to energy following the screening of trajectories was carried out, and the data were analyzed followed by the results (Pinto et al., 2019).

**TABLE 1:** Screening of lipases, amino acids corresponding to the respective active sites, and PDB.

Lipase	Cost ($)	PDB	Active site amino acid	Reference
*Candida antarctica B* (pH 7,4; 60°C)	$1,060.00 (250 mg)	5A6V	Ser 105, Asp 187, His 224	Cambillau et al. (1996); Kim et al. (2016); Figueiredo et al. (2019); Rabbani et al. (2015); Torres et al. (2008)
			Ser 105, Asp 187, His 224	
			S 105, H 224, D 187	
*Thermomyces lanuginosus* (pH 6.5; 50°C)	$529.00 (50 g)	5AP9	Ser 146, His 258, Asp 201	Tong et al. (2016); Skjold-Jørgensen et al. (2014); Singh et al. (2003)
			Ser 146, His 258, Asp 201	
*Burkholderia cepacia* (pH 7; 50°C)	$70.10	2NW6	S 87, D 264, H 286	Barbe et al. (2009); Santana et al. (2020); Lisboa et al. (2018)
			Ser 87, Asp 264, His 286	
*Candida rugosa* (pH 7,5; 45°C)	$363.00 (100 g)	3RAR	Ser 209, Glu 341, His 449	Santana et al. (2019); Vanleeuw et al. (2019); Cabrera-Padilla et al. (2012)
			Ser 209, His 449, Glu 341	
*Rhizopus niveus* (pH 7.4; 37°C)	$193.00 (50 g)	1LGY	Ser 45, Asp 204, His 257	Kohno et al. (1996); Rabbani et al. (2012); Alam et al. (2015)
			Ser 145, Asp 204, His 257	
Porcine pancreas (pH 8.9; 35°C)	$118.00 (1 mg)	1ETH	Ser 153, Asp 177, His 264	Guimarães et al. (2018); Hou et al. (2020); Goswami et al. (2012)
			Ser 153, Asp 177, His 264	
*Rhizomucor miehei* (pH 8; 45°C)	$529.00	3TGL	Ser 144, His 257, Asp 203	Brogan et al. (2014); Huang et al. (2014); Zhang et al. (2008)
			Ser 144, His 25, Asp 203	
*Photobacterium lipolyticum* (pH 8; 30°C)	-	2ORY	Ser 174, Asp 236, His 312	Joseph et al. (2007); Ryu et al. (2006)
			Ser 174, Asp 236, His 312	
Human gastric (pH 4; 37°C)	-	1HLG	Ser 153, His 353, Asp 324	Sams et al. (2016); Moreau et al. (1988)

The prices of biocatalysts were according to Sigma-Aldrich^®^ (https://www.sigmaaldrich.com/US/en).

**TABLE 2:** SMILES codes for binders and products, molecular formula, and structure of the substances applied in this study.

Substance	Canonical SMILES	Molecular formula	Structure
α-Tocopherol	CC1=C2C(O[C@@](C)(CC2)CCC[C@@H](CCC[C@@H](CCCC(C)C)C)C)=C(C(C)=C1O)C	C_29_H_5_O_2_	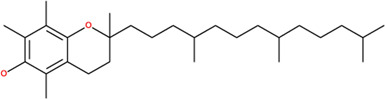
Vinyl acetate	C=COC(C)=O	C_4_H_6_O_2_	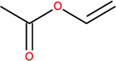
Vinyl nicotinate	C=COC(=O)C1=CN=CC=C1	C_8_H_7_NO_2_	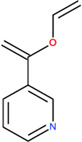
Vinyl succinate	C=COC(=O)CCC(=O)O	C_6_H_8_O_4_	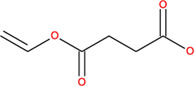
Vinyl ferulate	COC1=C(C=CC(=C1)C=CC(=O)OC=C)O	C_12_H_12_O_4_	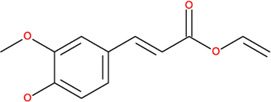
α-Tocopherol acetate	CC1=C2C(O[C@@](C)(CC2)CCC[C@@H](CCC[C@@H](CCCC(C)C)C)C)=CC(C)=C1OC(C)=O	C_30_H_50_O_3_	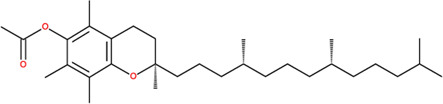
α-Tocopherol nicotinate	CC1=C(C(=C(C2=C1OC(CC2)(C)CCCC(C)CCCC(C)CCCC(C)C)C)OC(=O)C3=CN=CC=C3)C	C_35_H_53_O_3_	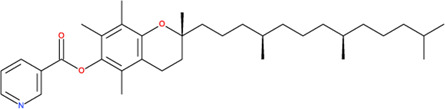
α-Tocopherol succinate	CC1=C(C(=C(C2=C1OC(CC2)(C)CCCC(C)CCCC(C)CCCC(C)C)C)OC(=O)CCC(=O)O)C	C_33_H_54_O_5_	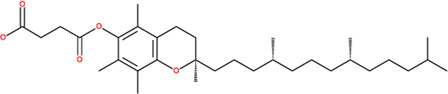
α-Tocopherol ferulate	CC1=C(C(=C(C2=C1OC(CC2)(C)CCCC(C)CCCC(C)CCCC(C)C)C)OC(=O)C=CC3=CC(=C(C=C3)O)OC)C	C_39_H_58_O_5_	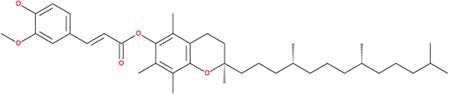

After that, the structure of each lipase, reaction analysis, transport of each molecule in the biocatalyst, behavior of ligands in the tunnels, and E_max_ and E_a_ as well as bottleneck radius (Å), length (Å), curvature, and throughput were analyzed according to the web server CaverWeb. Thus, to understand in a computational way the behavior of ligands in the tunnels present in the lipase structures is of great interest for biocatalysis because it allows the understanding of the reaction possibilities between the substrate and enzyme. The simulation was started with the identification of the number of tunnels and their properties. Then, the SMILES codes of the respective ligands were added, and the server calculated the energies [E_bound_, E_max_, E_surface_, E_a_, and ΔEBS (E_surface_) (Kcal/mol)] for the screening of trajectories in the tunnels. Based on this observation, data analysis was performed, and thus we predicted which tunnel was most likely to transport the substrates and products (Stourac et al., 2019).

**SCHEME 1:**
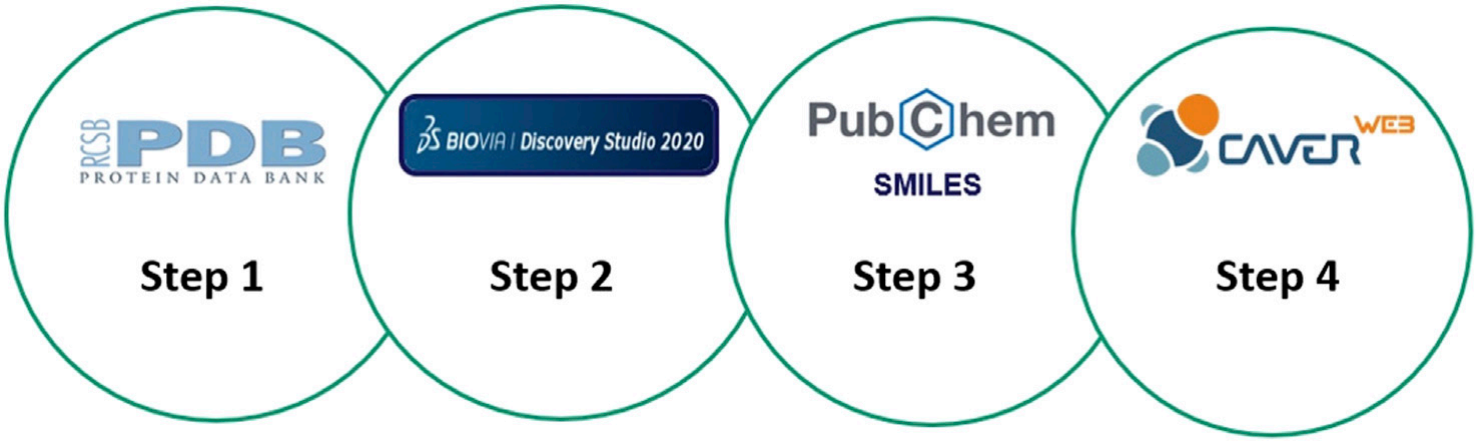
Representative scheme of the steps that were performed in the computational analysis. Step 1: Obtaining the PDB’s of the analyzed enzymes in the Protein Data Bank. Step 2: Structuring and identifying the enzymatic structure as well as identifying the active site of each enzyme. Step 3: Search and selection of the binders and products (SMILES codes) of this analysis. Step 4: Inserting the SMILES codes of the substances into the analyzed enzymes using the CaverWeb server.

The energy expenditure of each path is transformed into a new measure of the importance of a direction which is known as throughput. The values obtained by the transfer rate can vary between 0 and 1, and the closer the rate to 1, the greater the probability of transporting binders in the tunnel. However, tunnels that have a lower throughput have lower possibilities of transporting (Chovancova et al., 2012; Vavra et al., 2019). The possibilities of transporting binders in tunnels can be analyzed using E_max_ (highest binding energy in the path) and E_a_ (activation energy of the E_max_ association—E_surface_ for binders) and that the lower the values of E_max_ and E_a_, closer to 0, the greater the probability that a ligand has to go through the tunnel to reach the active site. Thus, to identify which tunnel is most likely to be transported, the morphological characteristics of the tunnels obtained in CaverWeb were observed along with the E_a_ and E_max_ energy values (Pinto et al., 2019; Vavra et al., 2019; Singh et al., 2021). A scheme of steps about this computational analysis is visualized in Scheme 1.

## Results

In *in silico* analysis of biomolecules, mainly lipases can be applied to verify enzymatic behavior under optimal conditions not including interference from temperature changes (Table 1), pressure, or immobilization techniques that can promote the best enzymatic performance in addition to being a low tool cost and easy access for the scientific community. Molecular docking is a tool used to estimate the chemical and biological activities of molecules, employing computational calculations prior to experimental processes and that demonstrates the energy parameters of the ligand molecules. The numerical values of these parameters are applied to compare the biological activities of mottraslecules against interactions between molecules and enzymes (Taslimi et al., 2020). Among the most different applications of molecular dynamics, the study of enzymatic tunnels and their analysis of substance transport can be highlighted for a better understanding of biocatalytic processes.

### Tunnel Analysis

**FIGURE 1:**
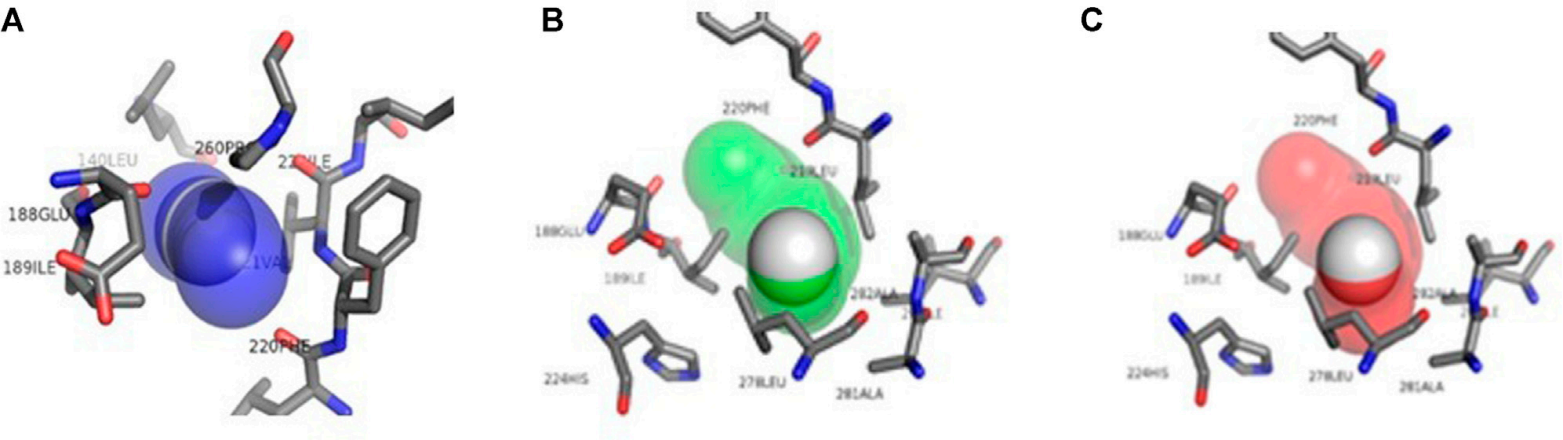
Structural representation of the amino acids present in the CALB access tunnels. Tunnel 1 in blue, tunnel 2 in green and tunnel 3 in red taken from CaverWeb (https://loschmidt.chemi.muni.cz/caverweb/) with PDB 5A6V.

The structural information that could be observed through CaverWeb was the binder inlet diameter (Å), tunnel length (Å), curvature, and the possibility of transporting through each tunnel present. This information is important to molecular dynamics, as it demonstrates the possibility of observing which pathway is most likely to enter the ligand to pass through the lipase structure and reach the active site to carry out the reaction of interest as well as demonstrate along the way that the binder can go through each tunnel. Each enzymatic tunnel has its own characteristic (Figure 1), not being the same tunnel with the same morphological or functional structure in the most different existing enzymatic structures. Thus, the conformational study of tunnels present in enzymes has great relevance to biocatalytic reactions, as it promotes the optimization of several enzymatic properties (Vavra et al., 2019).

**FIGURE 2:**
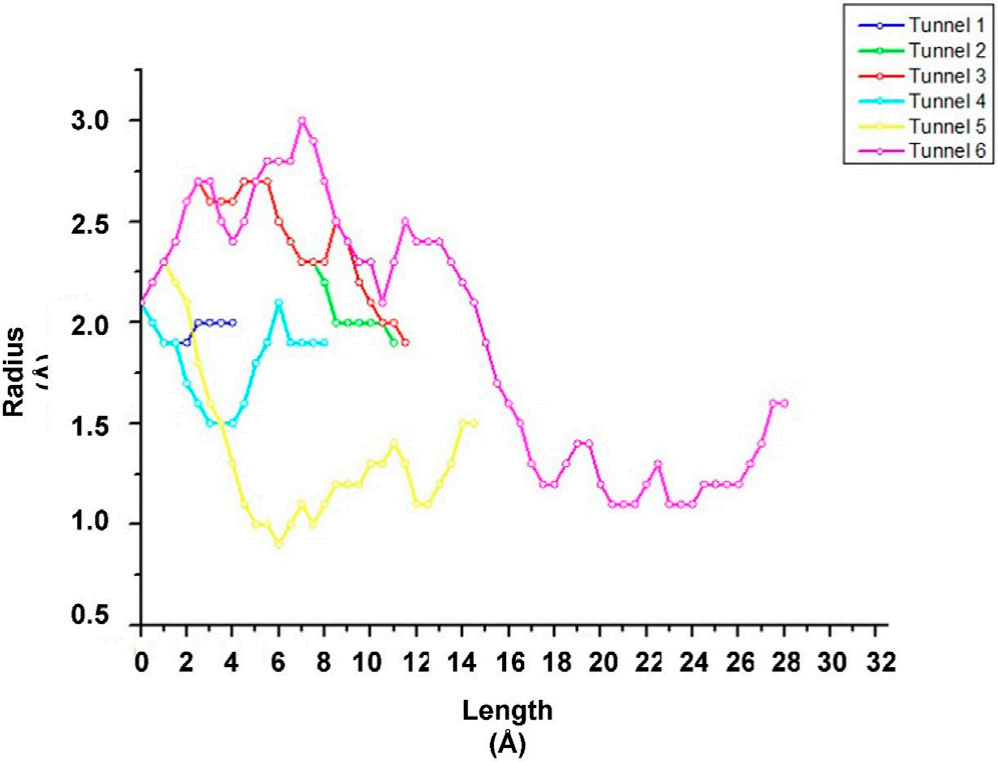
Graphic representation of the length and radius of tunnels 1 (blue), 2 (green), 3 (red), 4 (light blue), 5 (yellow) and 6 (pink) present in the CALB structure.

**FIGURE 3:**
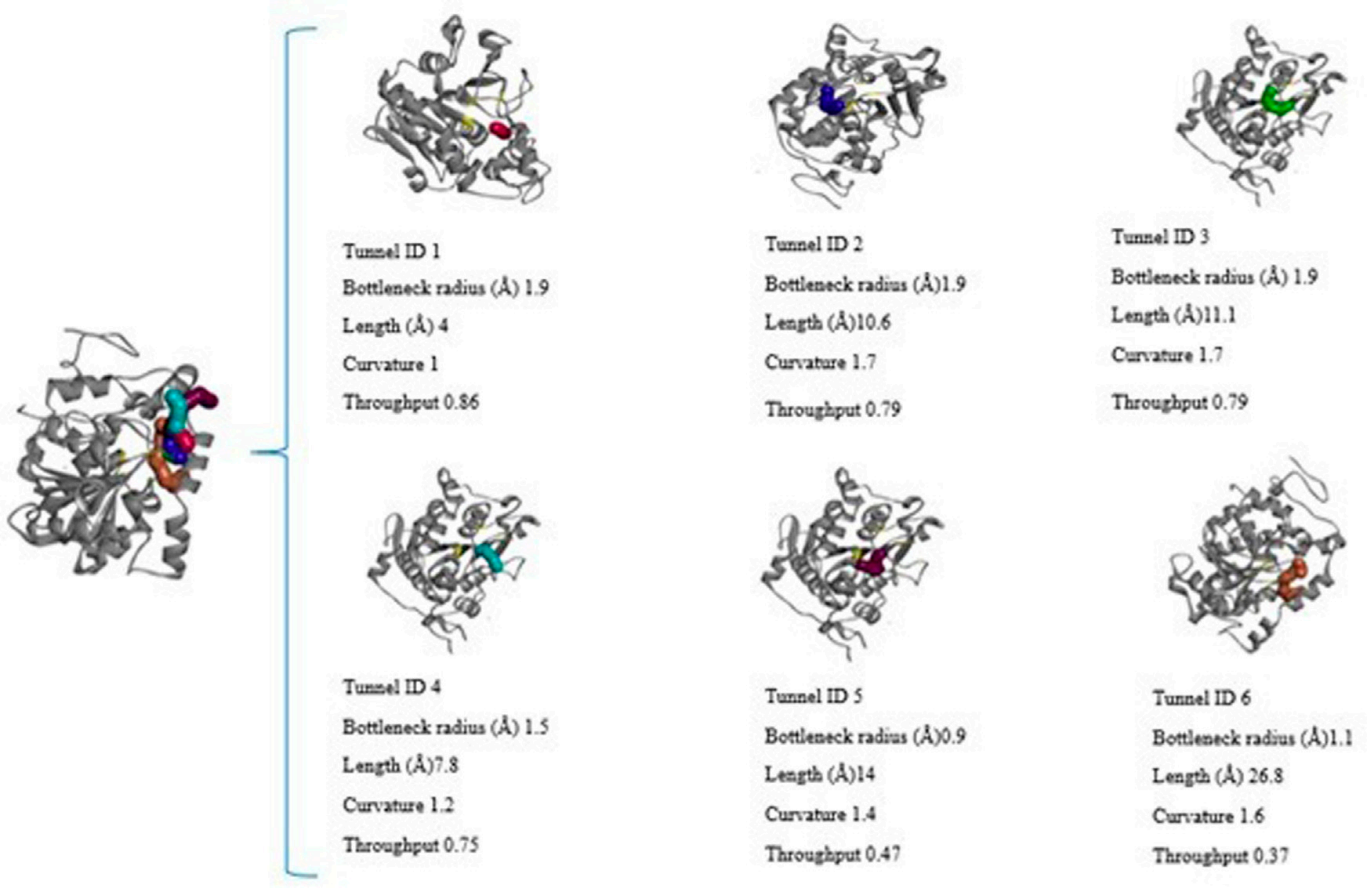
Graphical representation of the CALB crystallographic structure (PDB: 5A6V) in gray, in yellow the location of the active site, and the visualization of the 6 access tunnels in different colors that compose it, obtained in Caver Web and visualized in Discovery Studio, and the characteristics of tunnel size (Å), Length (Å), curvature, and the throughtput (possibility of transport).

To analyze the transport possibilities of the tunnels, it is necessary to understand the information about the energies that are demonstrated in CaverWeb (Figure 2). The energy values [E_bound_, E_max_, E_surface_, E_a_, and ΔEBS (kcal/mol)] that were obtained through the *in silico* analysis of the binders (α-tocopherol, vinyl acetate, vinyl succinate, vinyl nicotinate, and vinyl ferulate) and donated products (α-tocopherol acetate, α-tocopherol succinate, α-tocopherol ferulate, and α-tocopherol nicotinate) were verified about the tunnels present in the enzymatic structures individually (Figure 3). The lower absolute affinity energy (E_a_) value indicates that the binder has greater ease of substrate entry. ΔEBS corresponds to the energy difference between the active site present in the enzyme and its surface. Minimum bonding values, corresponding to the ligand that is connected to the active site and the surface, are related to the ligand that is connected to the surface (Pinto et al., 2019; Stourac et al., 2019). Substance transport analyses were performed with the 9 selected lipases. Consequently, it can be verified that *Thermomyces lanuginosus* lipase has 8 tunnels, among them only 2 were with high ligand transport probabilities that α-tocopherol presented a higher probability of entry through tunnel 1 with E_max_ −4.7 (kcal/mol) and E_a_ 0 (kcal/mol). The vinyls (vinyl acetate, vinyl nicotinate, vinyl succinate, and vinyl ferulate) were better transported through tunnel 2, and all products obtained were delivered through tunnels 1 and 2. *Burkholderia cepacia* lipase, despite having 4 tunnels, only tunnels 1 and 2 demonstrated to have transport probability, since α-tocopherol transport can be verified through either tunnel 1 or tunnel 2 [E_max_ 16.9 (kcal/mol); E_a_ 21.5 (kcal/mol) and E_max_ 25.4 (kcal/mol); E_a_ 17.7 (kcal/mol), respectively]. Only two vinyls have tunnel 1 entry, vinyl acetate and vinyl succinate with E_max_ −1 (kcal/mol) and E_a_ 1.9 (kcal/mol) and E_max_ −1.4 (kcal/mol) and E_a_ 2.6 (kcal/mol), respectively. For product output, only tunnel 1 was possible with α-tocopherol acetate [E_max_ −0.5 (kcal/mol) and E_a_ 1.8 (kcal/mol)] and α-tocopherol succinate [E_max_ 10.9 (kcal/mol) and E_a_ 0 (kcal/mol)].

For *Candida rugosa* lipase, there is a quantitative of 8 tunnels; however, tunnels 1 and 2 showed higher rates of substance transfer, though all substances showed entry and exit through tunnel 1, both ligands and products. *Rhizopus niveus* lipase has 4 tunnels in its structure, but only tunnels 1 and 2 are more likely to be transported. The tunnel with the better entry to α-tocopherol is tunnel 2 [E_max_ −4 (kcal/mol); E_a_ 0 (kcal/mol)], and it has lower values compared to tunnel 1 [E_max_ −3.9 (kcal/mol); E_a_ 0.3 (kcal/mol)]. The better entrance tunnel for all vinyls is tunnel 1, as it has smaller E_max_ and E_a_ closer to 0 (kcal/mol). For the exit of products, α-tocopherol acetate has been shown to exit both in tunnel 1 [E_max_ −4 (kcal/mol); E_a_ 0.2 (kcal/mol)] and in tunnel 2 [E_max_ −4.1 (kcal/mol); E_a_ 0.1 (kcal/mol)]. While α-tocopherol succinate exits through tunnel 1 [E_max_ −4.1 (kcal/mol); E_a_ 0 (kcal/mol)], α-tocopherol ferulate exits through tunnel 2 [E_max_ −4 (kcal/mol); E_a_ 0.1 (kcal/mol)] and α-tocopherol nicotinate through tunnel 2 [E_max_ −4.2 (kcal/mol); E_a_ 0 (kcal/mol)]. Porcine pancreas lipase has a quantitative of 39 tunnels in its structure, with 11 tunnels having a bigger probability to be transported in its structure. For α-tocopherol, the better entrance tunnel was tunnel 4, with E_max_ −4.8 (kcal/mol) and E_a_ 0 (kcal/mol). For the other binders such as vinyl acetate [E_max_ 02.1(kcal/mol) and E_a_ 0.1 (kcal/mol)], vinyl succinate [E_max_ −2.8 (kcal/mol) and E_a_ 0 (kcal/mol)], vinyl ferulate [E_max_ −4.2 (kcal/mol) and E_a_ 0 (kcal/mol)], and vinyl nicotinate [E_max_ −3.4 (kcal/mol) and E_a_ 0.2 (kcal/mol)], better entry was through tunnels 12, 1, 4, and 12, respectively. For α-tocopherol acetate, the best tunnel was 4 with E_max_ −4.6 (kcal/mol) and E_a_ 0 (kcal/mol). For α-tocopherol succinate, the better tunnel was 4 with E_max_ −4.8 (kcal/mol) and E_a_ 0 (kcal/mol). For α-tocopherol ferulate, the better tunnel was 1 with E_max_ −4.8 (kcal/mol) and E_a_ 0 (kcal/mol). For α-tocopherol nicotinate, the better tunnel was 6 with E_max_ -5 (kcal/mol) and E_a_ 0.6 (kcal/mol).

*Rhizomucor miehei* lipase has a quantitative of 3 tunnels in its structure, and the 3 tunnels were with transport probabilities. For α-tocopherol, the tunnel with the highest probability of entry is tunnel 1 [E_max_ −4.5 (kcal/mol); E_a_ 0.2 (kcal/mol)]. For the acetate, succinate, and nicotinate vinyl substrates, the tunnel with the highest probability of entry is tunnel 1 [E_max_ −2.2 (kcal/mol); E_a_ 0 (kcal/mol), E_max_ -2.9 (kcal/mol); E_a_ −0.1 (kcal/mol), and E_max_ −3.4 (kcal/mol), E_a_ 0.1 (kcal/mol), respectively} while vinyl ferulate with E_max_ −4 (kcal/mol) and E_a_ 0 (kcal/mol). The output of the products of interest can be verified that tunnel 1 was the tunnel with the highest probability of output for α-tocopherol acetate [E_max_ −4.2 (kcal/mol); E_a_ 0 (kcal/mol)], α-tocopherol succinate [E_max_ −4.3 (kcal/mol); E_a_ 0.5 (kcal/mol)], α-tocopherol ferulate [E_max_ −4.2 (kcal/mol); E_a_ 0.3 (kcal/mol)], and α-tocopherol nicotinate [E_max_ −4; E_a_ 0.8 (kcal/mol)]. These results demonstrate that the products of interest leave through the same tunnel as the ligands.

*Photobacterium lipolyticum* lipase has in its structure 5 tunnels with only 2 with greater transport probability (1 and 2). While α-tocopherol showed entry only through tunnel 2 with E_max_ −0.4 (kcal/mol) and E_a_ 1.6 (kcal/mol), vinyl acetate [E_max_ −2.6 (kcal/mol); E_a_ 0 (kcal/mol)], vinyl succinate [E_max_ −3.7 (kcal/mol); E_a_ 0 (kcal/mol)], vinyl ferulate [E_max_ −5.1 (kcal/mol); E_a_ 0.1 (kcal/mol)], and vinyl nicotinate [E_max_ −4.2 (kcal/mol); E_a_ 0 (kcal/mol)] showed entry through tunnel 1, and only α-tocopherol acetate showed exit through tunnel 1 [E_max_ −4.2 (kcal/mol); E_a_ 0 (kcal/mol)]. Human gastric lipase has 7 tunnels in its structure; however, only 2 were with greater possibilities for transporting substances. α-Tocopherol demonstrated entry into tunnels 1 and 2 with E_max_ 11.4 (kcal/mol)and E_a_ 8.2 (kcal/mol) and E_max_ 15.3 (kcal/mol) and E_a_ 8.6 (kcal/mol), respectively. Only vinyl acetate [E_max_ −2.7 (kcal/mol); E_a_ 8.2 (kcal/mol)] and vinyl succinate [E_max_ −3.3 (kcal/mol); E_a_ 0.7 (kcal/mol)] were verified for transport in tunnel 1, while vinyl ferulate and vinyl nicotinate were not visualized. For the output of the products of interest in this lipase, it can be verified that only tunnel 1 showed the possibility of transport for both α-tocopherol acetate [E_max_ 6.8 (kcal/mol); E_a_ 11.3 (kcal/mol)] and α-tocopherol succinate [E_max_ 7.6 (kcal/mol); E_a_ 12 (kcal/mol)].

**FIGURE 4:**
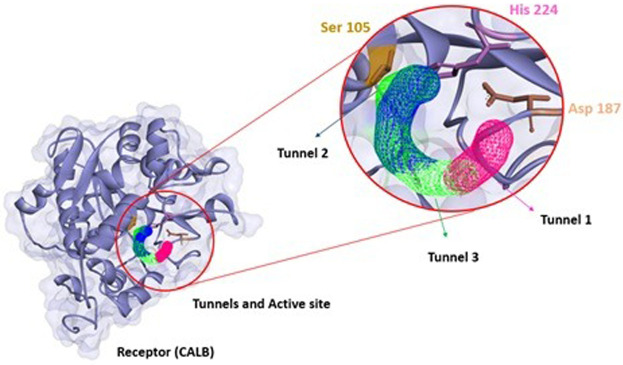
Structure of CALB (PDB: 5A6V) with tunnels (tunnel 1 pink, tunnel 2 green, and tunnel 3 blue), and the respective active site (Ser 105, Asp 187 and His 224) calculated using CaverWeb and identificated using Discovery Studio.

Thus, understanding the behavior of a ligand regarding the enzymatic structure is important to analyze the path that these molecules take to perform the biocatalysis. Figure 4 represents the conformational structure of CALB where the 3 tunnels (analyzed in the CaverWeb) were with the highest probability of transporting substances, confirming the experimental results obtained by Torres et al. (2008). CALB is a biocatalyst that realizes great catalytic reactions such as esterification (Santin et al., 2014), transesterification (Ferrer et al., 2005), acetylation (Torres et al., 2008), and others can be observed due to their morphological characteristics. To understand how the transport of molecules through enzymatic tunnels works, it is important to study molecules and substances through enzymatic tunnels.

### Substrate Transport

To catalyze a substrate, it is necessary to understand the following points: the substrate must fit in the binding tunnel present in the enzyme, and the substrate must be able to pass through the tunnel. In enzymatic structures, the active site is located in the internal cavity, while the tunnels are connections between the active site and the substrate (Wang et al., 2021). Lipases are a family of enzymes that contain a tunnel region that connects the acyl end of their substrates. They have a length of 8–22 Å and a geometry that can vary greatly between different lipases. By sketching the tunnel wrap residues, specificity for the length of the acyl chain can be achieved (Kokkonen et al., 2019).

To consist how substrate transport happens, the ligand is transported toward or away from the active site within the receptor through a tunnel. However, each receiver can have multiple tunnels that lead to the same active location. In CaverWeb, the active site is considered as the beginning of the tunnel and the surface of the receiver as the end of the tunnel. The calculation of the tunnels starts by selecting the starting point at the active site through energies, where the minimum size of the probe defines the minimum radius that the tunnel must have to be found. The depth and radius of the tunnel define the surface of the substrate in the receiver (Hozzová et al., 2021).

For the energetic understanding regarding the substrates submitted to CALB, Table 3 shows the input energies of the substrates in the main access tunnels. For α-tocopherol, the best entry tunnel was tunnel 3 with E_max_ −4.6 and E_a_ 1.1. Vinyl acetate showed very close entry possibilities in the 3 tunnels (tunnel 1: E_max_ −2.4 and E_a_ 0.1; tunnel 2: E_max_ −2.6 and E_a_ 0; and tunnel 3: E_max_ −2.5 and E_a_ 0.1) which indicates that this ligand has different input possibilities.

**TABLE 3:** Information on the energies corresponding to the input substrates in the tunnels with the highest probability in CALB.

	Tunnel 1		Tunnel 2		Tunnel 3	
	E_max_	E_a_	E_max_	E_a_	E_max_	E_a_
α- Tocopherol	−1.9	3	−4.2	1.6	−4.6	1.1
Vinyl acetate	−2.4	0.1	−2.6	0	−2.5	0.1

**FIGURE 5:**
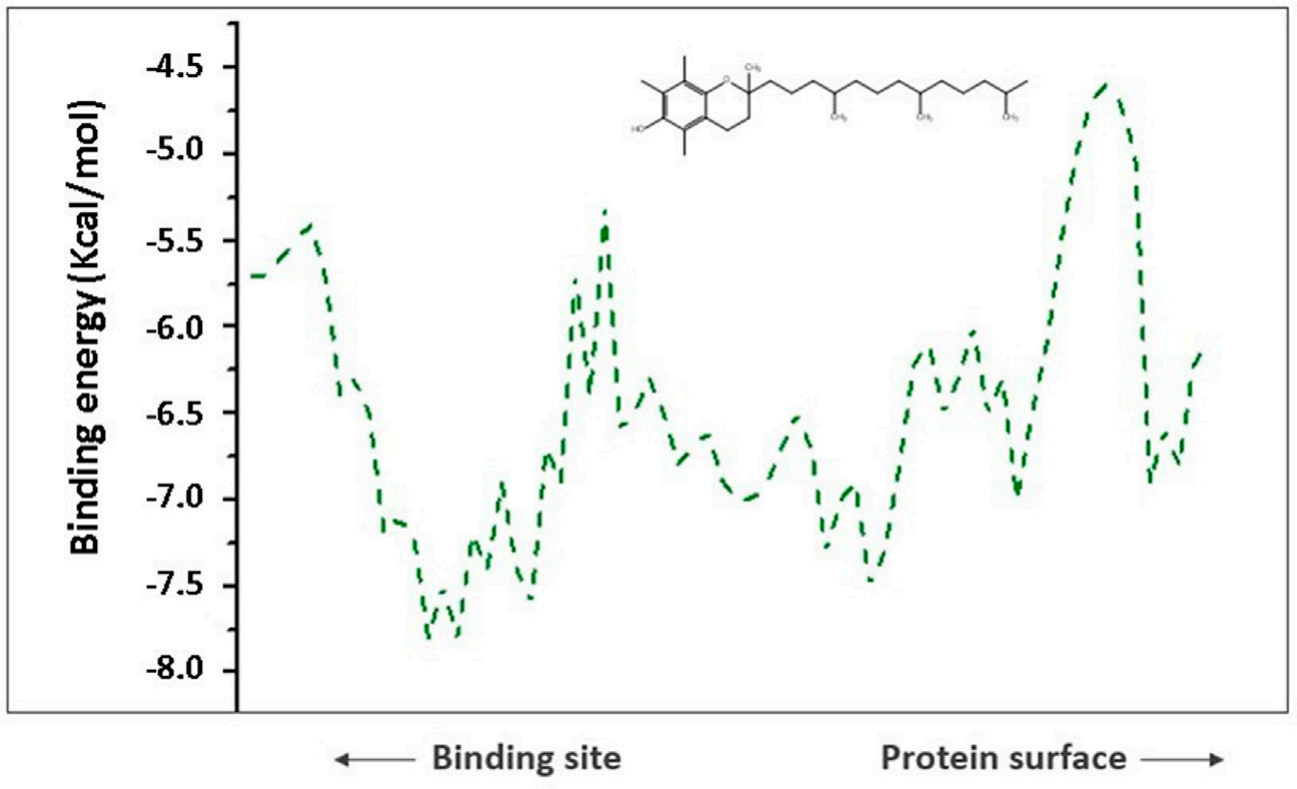
Representation of binding energies, trajectory and tunnel radius for α-tocopherol in tunnel 3.

**FIGURE 6:**
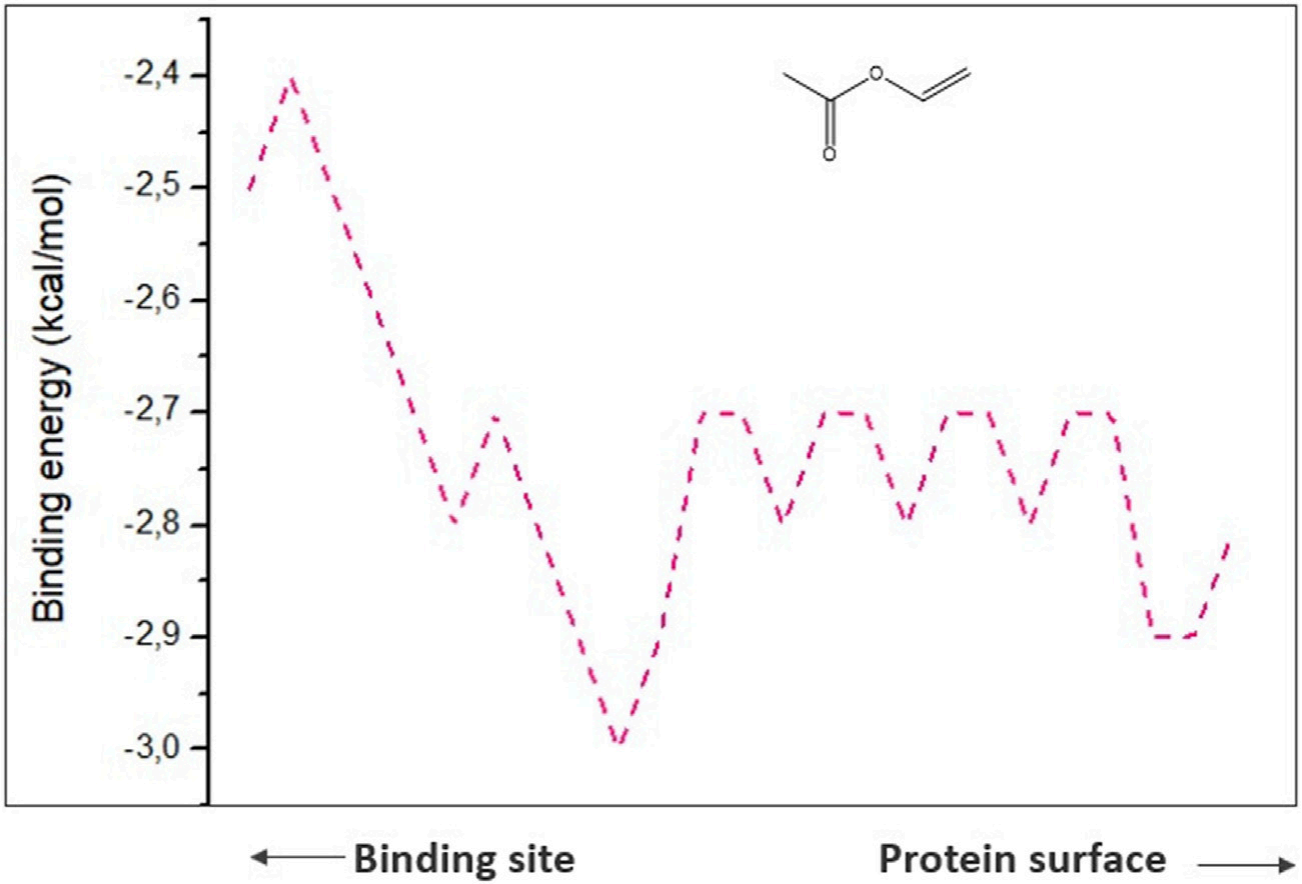
Representation of binding energies, trajectory and tunnel radius to vinyl acetate referring to tunnel 1.

**FIGURE 7:**
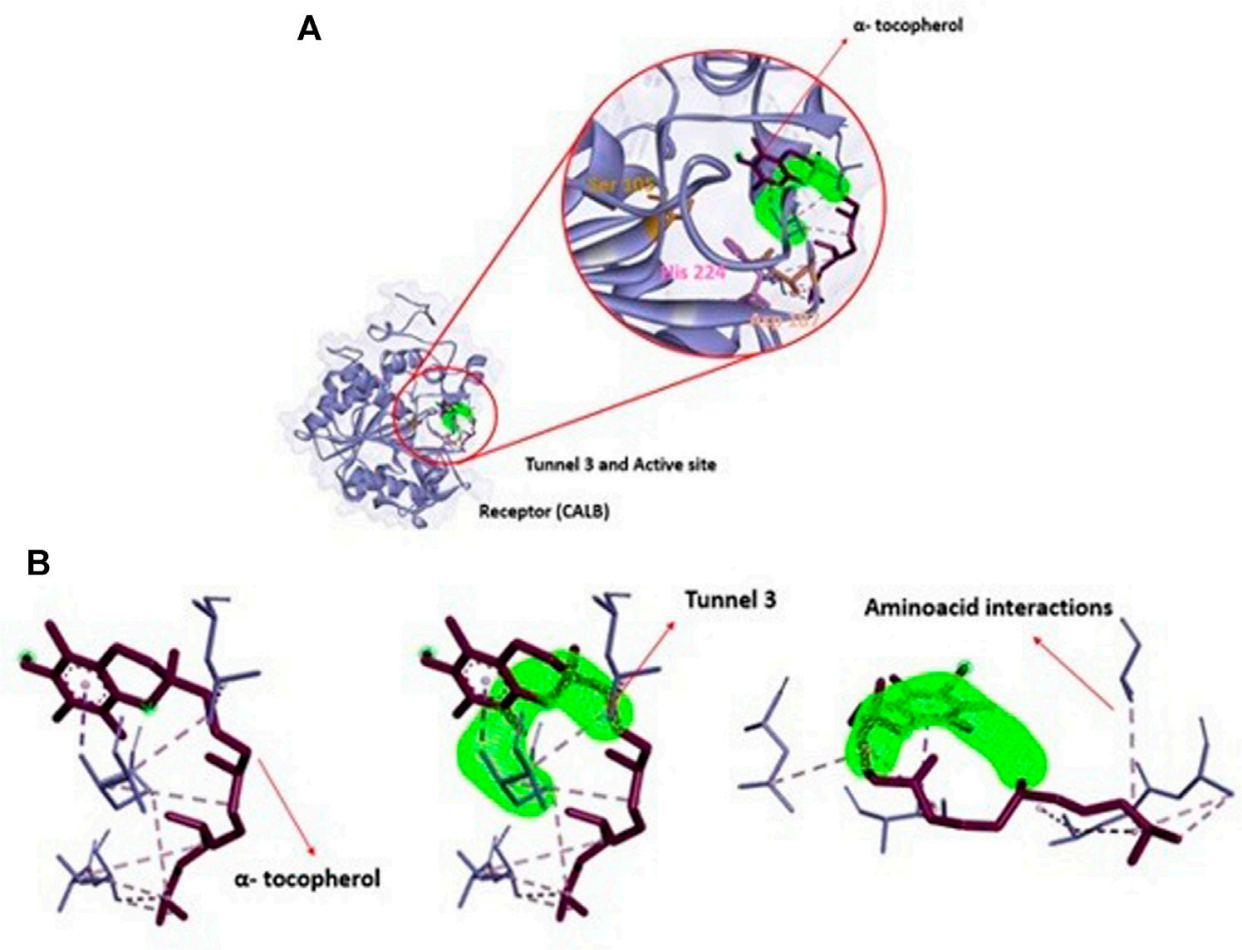
**(A)**: Structural representation of receptor (CALB), active site (Ser 105, Asp 187 and His 224) in relation to position of tunnel 3 and the ligand (α-tocopherol). **(B)**: View of ligand α-tocopherol (purple) in tunnel 3 (green) apllied Caver Web in protein.

To comprehend analysis of the transport of substances or ligands in a specific tunnel, as we can see in Figure 5, the representation of the energy trajectory of α-tocopherol through the tunnel with the highest transport probability was represented by tunnel 3. In Figure 6, the vinyl acetate energy trajectory was through the tunnel with the highest probability of transport (tunnel 1). In both images, the direction of the protein surface as well as the direction of the binding site can be seen. α-Tocopherol, through its structural conformation in the best transport probability tunnel, as well as the interactions with the amino acids that are disposed there can be visualized in Figures 7A,B.

To get the interactions of vinyl acetate with the protein (Table 4), it is observed that the existing interactions are van der Waals interactions, which demonstrate an interaction, even if weak, with the amino acids Leu 140, Ile 189, and Glu 188. It is important to highlight that van der Waals interactions play an important role in structure formation, energetic stability, and reaction mechanisms for a wide variety of molecules (Kleshchonok and Tkatchenko, 2018).

**TABLE 4:** Amino acids and interactions type around the corresponding tunnel (tunnel 1) and ligand (vinyl acetate).

Substance	Amino acid	Interaction
Vinyl acetate	Leu 140	van der Waals
	Ile 189	
	Glu 188	

Therefore, obtaining knowledge of the transport of ligands in the access tunnels of a protein structure as well as understanding the types of bonds that the ligands have with the respective amino acids that are disposed of there is of fundamental importance.

### Product Transport

Through the energetic information of the substrates that was obtained, the characteristics of the 3 tunnels present the greatest possibilities of the output of the product (α-tocopherol esters). The information presented regarding the conformational study of the tunnels present in CALB is considered of primordial importance, as it is through these characteristics that we can understand the energetic behavior of binders and products.

**FIGURE 8:**
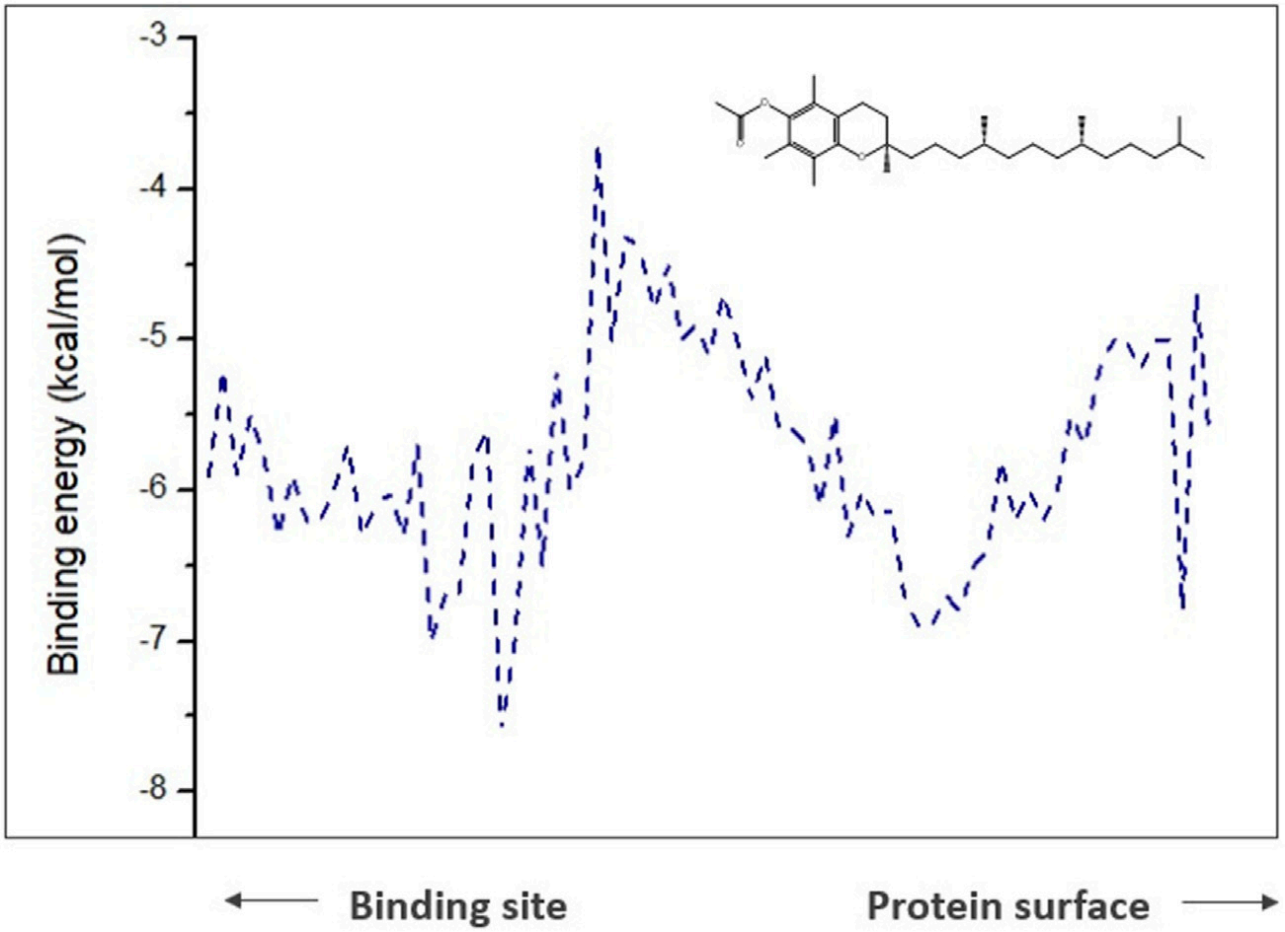
Representation of the trajectory binding energies and ray size for α-tocopherol acetate in tunnel 2.

However, for the products analyzed in this research, the 4 products showed transport potential through the first 3 tunnels located in CALB but with a greater possibility of exit through tunnel 2 (Figure 8) which were α-tocopherol acetate with E_max_ −3.7 kcal/mol and E_a_ 2 kcal/mol, α-ferulate tocopherol with E_max_ −4.7 kcal/mol and E_a_ 1.9 kcal/mol, and α-tocopherol nicotinate with E_max_ −4.7 kcal/mol and E_a_ 1.9 kcal/mol and through tunnel 3 was α-tocopherol succinate with E_max_ −4.8 kcal/mol and E_a_ 1.8 kcal/mol. It is important to note that although α-tocopherol acetate is the most commonly used ester in the pharmaceutical and cosmetic industry, α-tocopherol succinate, α-tocopherol nicotinate, and α-tocopherol ferulate are of fundamental importance for biological studies and biotransformation, as they are also addressed in the literature (Xin et al., 2011; Jiaojiao et al., 2021).

Molecular dynamics tools can perform an orientation that obtains a higher probability of one or more molecules in the active sites of the lipase. As molecular docking will generate a series of possible complexes, the best orientation will be selected through scoring functions and used to predict binding affinity. However, it is clear that there are different tunnels present in the lipase, and Table 5 shows the summary of data for the tunnels in the target lipase. Based on information about the structure of biomolecules and prior knowledge of ligands, the application of molecular dynamics tools to predict their behavior and optimize future reaction processes is of fundamental importance.

**TABLE 5:** Characteristics related to CALB tunnels with more probability to realize the transport of products.

Substance	Tunnel 1		Tunnel 2		Tunnel 3	
	E_max_	E_a_	E_max_	E_a_	E_max_	E_a_
	kcal/mol		kcal/mol		kcal/mol	
α-Tocopherol acetate	−2.9	3.9	−3.7	2	−4.2	2.7
α-Tocopherol succinate	−3.7	2.4	−4.4	1.8	−4.8	1.8
α-Tocopherol ferulate	-3	3	-4.7	1.9	-3.5	2.2
α-Tocopherol nicotinate	−4.3	2.7	−4.7	1.9	−4.5	1.3
Bottleneck radius (Å)	1.9		1.9		1.9	
Length (Å)	4		10.6		11.1	
Curvature	1		1.7		1.7	
Throughput	0.86		0.79		0.79	

**FIGURE 9:**
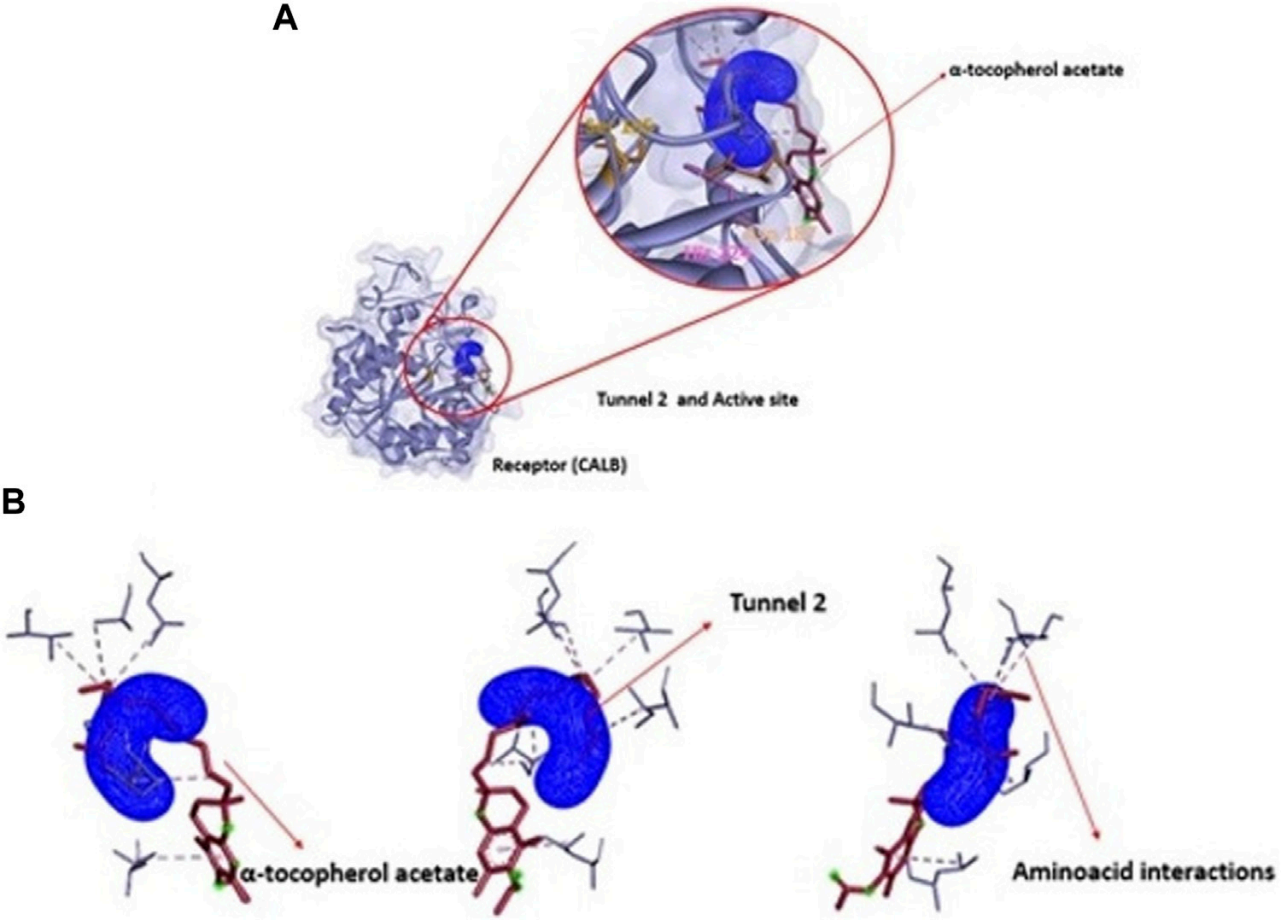
**(A)** Structural representation of receptor (CALB), active site (Ser 105, Asp 187 and His 224) in relation to position of tunnel 1 and the ligand (Vinyl acetate). **(B)**: View of ligand Vinyl acetate (blue) in tunnel 1 (pink) apllied Caver Web in protein.

To analyze the output of the product of interest, α-tocopherol acetate passes through an energy trajectory that can be seen in Figure 8. This representation demonstrates the energy profile through the molecular chain in tunnel 2, which is the tunnel with great possibility of passage of this substance. Figures 9A,B show the position of α-tocopherol acetate about the tunnel and the receptor (CALB) and the disposition of this substance about the exit tunnel of the product as well as the interactions of this molecule with the amino acids present in the enzymatic structure of CALB.

Table 6 shows the amino acids that interact with α-tocopherol acetate as well as the types of interaction that are related to this molecule. Amino acids Ala 279, Ala 282, Glu 188, Leu 140, Thr 138, and Thr 40 have van der Waals-type interactions. It can be verified that the interactions that exist are van der Waals interactions, which express an interaction of the substance, even if weak, with the amino acids present in the enzyme. These interactions play an important role in the formation of structures, energy stability, and reaction mechanisms for a wide variety of molecules (KLESHCHONOK; TKATCHENKO., 2018). The amino acid Leu 278, on the other hand, has a pi–alkyl-type interaction, and the amino acids Val 154, Ile 285, and Leu 144 perform alkyl-type interactions. Since van der Waals-type interactions are weaker interactions, the pi-alkyl and alkyl interactions are characterized by hydrophobic bonds.

**TABLE 6:** Amino acids and interactions type around the corresponding tunnel (tunnel 2) and ligand (α-tocopherol acetate).

Substance	Amino acid	Interaction
α-Tocopherol acetate	Ala 279	van der Waals
	Ala 282	
	Glu 188	
	Leu 140	
	Thr 138	
	Thr 40	
	Leu 278	Pi–alkyl
	Ile 189	Alkyl
	Val 154	
	Ile 285	
	Leu 144	

### Potential of Computational Analysis

**FIGURE 10:**
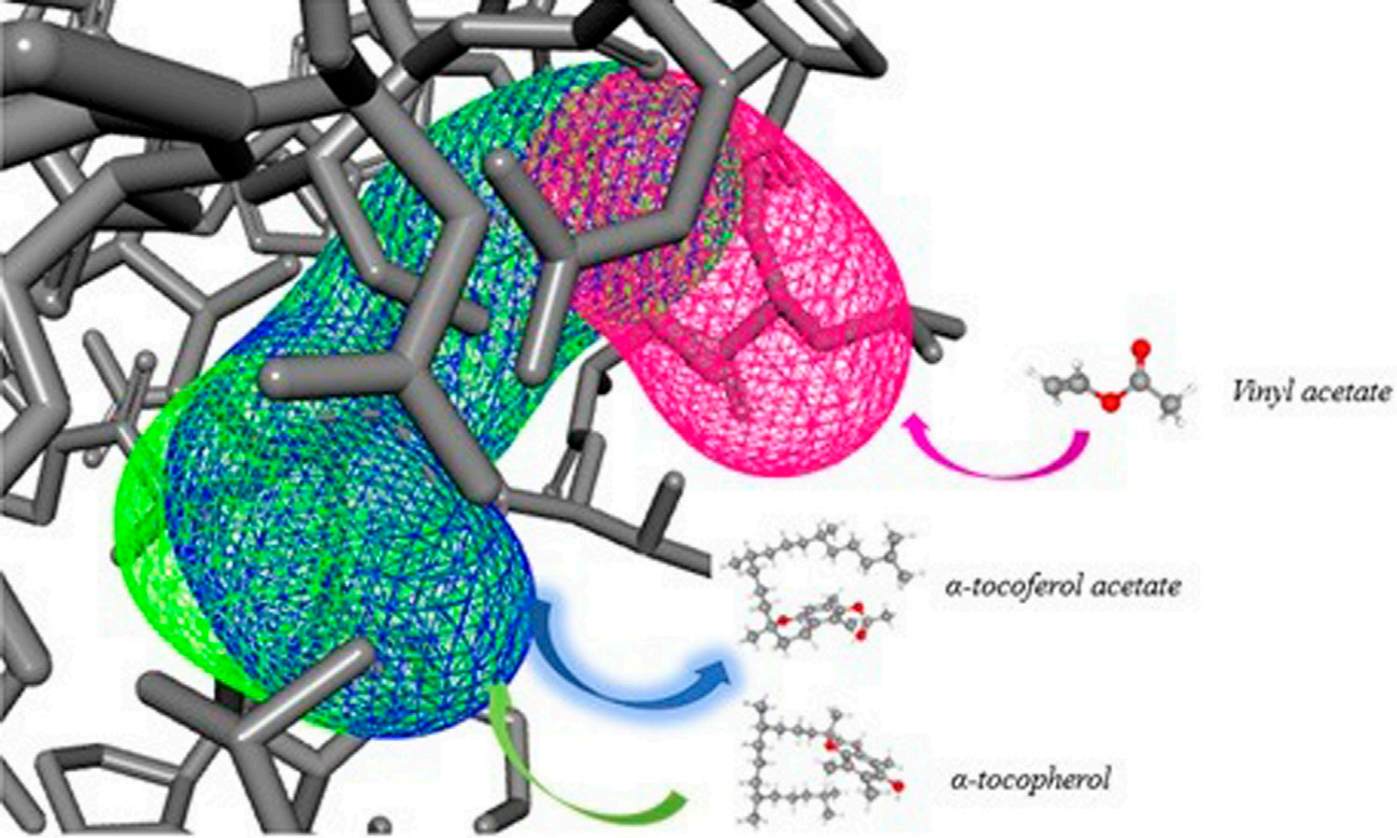
Representative diagram of the 3 most promising tunnels for transport in CALB, in pink (tunnel 1), blue (tunnel 2), and green (tunnel 3) and the demonstration of binder entry and exit of the product.

Figure 10 represents how the entry of ligands (α-tocopherol and vinyl acetate) occurs to obtain α-tocopherol acetate. The characteristics of CALB tunnels demonstrated in CaverWeb represent a particularity, which allows this lipase to have a significant reaction potential compared to other analyzed lipases.

Tunnel 1 has shorter lengths (Å) (4) and curvature (Å) (1); however, it has a higher efficiency (0.86). Tunnels 2 and 3, on the other hand, have the same throughput (0.79) and the same curvature (1.7), but tunnel 3 has a greater length (Å) (11.1) than that of tunnel 2 (10.6). The neck radius Å for the 3 tunnels was similar. What stands out in this analysis was the verification of the condescending behavior of the binders in the tunnels. Note that α-tocopherol enters through tunnel 3, vinyl acetate through tunnel 1, and the product (α-tocopherol acetate) exits through tunnel 2. This implies that demonstrating the passage of these substances through different tunnels denotes ease of entry of binders and exit from the product of interest.

## Discussion

### Tunnel Analysis

Access tunnels provide information about different important functions of enzymes, such as the distinction between substrates with higher transport probability, the control of the entry of co-substrates or solvent, and the observation of possible enzymatic damage caused by transition metals. as well as the prevention of cell damage by highly reactive intermediates and metabolites, the simultaneous order of individual catalytic steps, synchronization of reactions that require a large number of substrates, intermediates and/or cofactors and, finally, the spatial safety concentration of the reactant species (Kokkonen et al., 2019). This knowledge provides the obtention of transport interest (substrates and products) on tunnels.

According to Park et al. (2013), the substrate entrance is delimited by hydrophobic residues, but unlike other lipases, CALB does not have a cap and does not undergo interfacial activation. Four hydrophobic residues, Ile 189, Leu 278, Ala 282, and Ile 285, form a narrow tunnel that must be traversed by the substrate before the formation of the second tetrahedral intermediate. These amino acids can be observed in tunnels 1, 2, and 3 that make up the CALB represented in Figure 1.

Most tunnels function as extensions of the active site of an enzyme, which are characterized in their structural conformation by an entrance and exit, and also in the functioning of a natural connection between substances. The conformational characteristic and size of a tunnel can not only predict the maximum size that a ligand can pass but also inform the number of interactions that can be performed or the barriers that a ligand can face when being transported to the tunnel active location in an energy process (Kingsley and Lill, 2015).

Molecular dynamics analyses are used to calculate the binding affinities of the biomolecule to the ligand. These methods use an energetic approach to calculate the relative free energy binding between a receptor and two ligands based on the thermodynamic cycle between the molecules (Pinto et al., 2019). Thus, analyzing individually and particularly the structure of each tunnel present in the enzymatic structure (Figures 3, 4) is a fundamental importance to understand the transport possibilities that a ligand is subjected to energetically.

The active site is a specific space that is usually hidden in the protein, since the chemical reaction between the protein and the ligand can take place in this area. Consequently, the importance of access tunnels for these ligands is directly linked to the amino acids that are located around the tunnel, which substantially enable the structural and kinetic stability of the studied protein (Vonásek et al., 2019). Thus, most of the non-active sites reported so far are located in the flexible loop regions. According to their functionalities, the positions of non-active sites can be divided as follows: protein surface, region of turns, and the substrate tunnel, and the latter being considered an alternative approach to understand catalyst mechanisms and enzymatic engineering (Wang et al., 2021). Therefore, studying the transport of ligands in enzyme tunnels is of fundamental importance for biocatalysis.

### Substrate Transport

Thus, it is observed (Table 7) that the amino acids Ala 279, Leu 278, Ala 282, and Leu 140 have alkyl-type interactions with the α-tocopherol molecule. Ile 189, on the other hand, has a pi–sigma-type interaction, while amino acids Glu 188, Leu 144, Ala 141, Thr 138, Val 154, Gln 157, Thr 40, and Ile 285 have van der Waals-type interaction. Thus, binding orientations with ligands structurally related to binding sites can present a variety of interactions, including hydrophobic interactions (pi–sigma, alkyl, and pi-alkyl) as well as van der Waals-type interactions, and interactions of this type are characterized by being weaker compared to the other bonds represented here (Kleshchonok and Tkatchenko, 2018; Ranjbar et al., 2020; El Aissouq et al., 2021).

**TABLE 7:** Amino acids that α-tocopherol interacts with as well as the type of interaction corresponding to this ligand.

Substance	Amino acid	Interaction
α-Tocopherol	Ala 279	Alkyl
	Leu 278	
	Ala 282	
	Leu 140	
	Ile 189	Pi-–sigma
	Glu 188	van der waals
	Leu 144	
	Ala 141	
	Thr 138	
	Val 154	
	Gln 157	
	Thr 40	
	Ile 285	

According to Kumaresan et al. (2011), the activity and enantioselectivity of CALB are directly influenced by the interaction of the substrate with three binding pockets that are located close to the active site, being differentiated into 3 types with different constituent amino acids. Among them are medium pocket (G39, T42, S47, W104, and A225), large pocket (A141, L144, V154, I285, P289, and K290), and acyl (D134, T138, Q157, I189, and V190). From this understanding, it is possible to analyze some amino acids that α-tocopherol interacts with corresponding to the large pocket (Ala 141, Leu 144, Val 154, and Ile 285) as well as in the acyl pocket with the amino acids Ile 189 and Thr 138.

Ferrari et al. (2014) carried out a study based on the backbone protein and the other based on the oleoyl chain and the backbone residues (CALB) that constitute the catalytic cavity. Among them can be observed the amino acids: Thr 138, Ile 189, Val 190, Asp 134, Thr 40, Gln 157, Ile 285, Gly 44, and Val 149 as well as the residues that constitute the oxyanion hole: Thr 40 and Gln 106. Kim et al. (2014) identified that of all residues present in CALB, two isoleucines, ILE-189 and ILE-285, play a key role in the open-closed dynamics of the cavity and can hinder the passage of the substrate to the catalytic site. Consequently, it is believed that the positions of the amino acids that form the biocatalyst in its cavity influence the enzymatic activity, since the CALB catalytic cavity is divided into an acyl and alcohol group next to the ILE close to the cavity, and this conformation can affect the enzyme activity.

**FIGURE 11:**
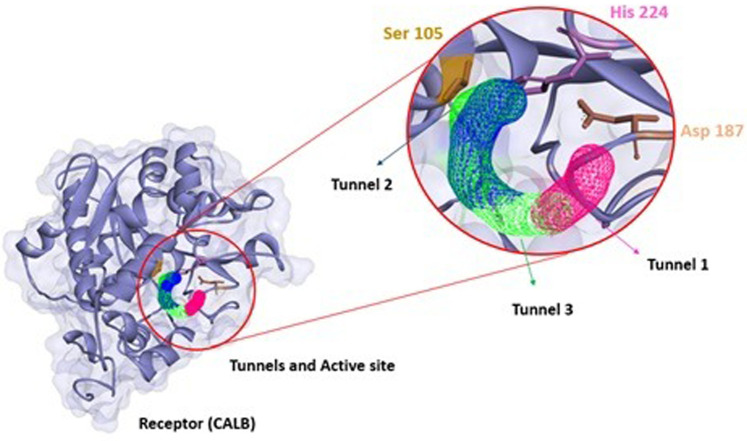
**(A)** Structural representation of receptor (CALB), active site (Ser 105, Asp 187 and His 224) in relation to position of tunnel 2, the ligand (α-tocopherol acetate) and **(B)** product α-tocopherol acetate (purple) in tunnel 2 (blue) apllied Caver Web in protein.

Therefore, performing the analysis of the ligands in the access tunnels of the substrates in the cavities present in the biocatalyst in relation to the amino acids present is important for the understanding of biocatalytic reactions. In Figures 11A,B, it is possible to observe the transport of vinyl acetate corresponding to tunnel 1 of CALB. Vinyl acetate is an acylating agent which undergoes nucleophilic attack by the amino group of an amino acid or peptide fragment that can result in the formation of a peptide bond between them (Machado et al., 2004; Barbosa et al., 2011).

### Product Transport

To analyze the functioning of the transport of substances about the interaction with the active site, Chaput et al. (2012) report that interactions of a substance with the side chains that are close to the active site can occur. Thus, it can be observed that in his work the amino acids that interacted with the ester under study were Thr 103, Gln 106, Thr 40, Thr 42, Ile 285, Ala 282, Leu 278, Ser 47, and Trp 104, corroborating some amino acids related to α-tocopherol acetate (Thr 40, Ile 285, Ala 282, and Leu 278).

For the output of CALB products, the best tunnel to exit α-tocopherol acetate (E_max_ −3.7 kcal/mol and E_a_ 2 kcal/mol) (Figure 6), α-tocopherol succinate (E_max_ −4.8 kcal/mol and E_a_ 1.8 kcal/mol), and α-tocopherol nicotinate (E_max_ −4 .5 kcal/mol and E_a_ 1.3 kcal/mol) is tunnel 3. Through the binders and products on the tunnels present in the CALB, it can be seen that the substrates’ entrance and exit tunnels are distinct, which denotes an optimized possibility of obtaining reaction products.

### Potential of Computational Analysis

For better knowledge of this mechanism, Kyriakou et al. (2012) carried out in their work a *in silico* study of how quercetin (a type of flavonoid) behaves in CALB to perform an acylation of this component. It can then be seen that this flavonoid can be favorably accommodated for transformation into the substrate-binding site of CALB.

Consequently, product formation was observed *in situ* with a yield of 45% only in the presence of the enzyme and optimal reaction conditions (solvent and acyl donor). The great interest also for biocatalytic processes is to consider the importance and high interest of biochemistry in given industrial applications. Molecular dynamics tools can be applied to shorten times in search of new ecologically correct routes; some studies using CALB are already known for their high efficiency and high selectivity applied in different industrial processes or suitable catalytic activities to enable biotransformation (Idris and Bukhari, 2012; Duncan and Suzuki, 2017). Thus, the biotransformation using CALB as a biocatalyst to obtain α-tocopherol acetate is already known, and the molecular dynamics tool showed that it was possible to understand the behavior of this biocatalyst through the substrate.

## Conclusion

Through the computational screening performed in this work, it can be verified that the number of existing tunnels in the enzymatic structure does not imply a better biocatalytic result, since the lipase with the largest number of tunnels submitted to *in silico* analysis in this work was the LPP with 39 tunnels, and no significant results were obtained. On the other hand, the lipase that best obtained the results was CALB (with 6 tunnels) because the characteristics of the tunnels present in its structure, compared to the binders submitted for analysis, were more likely to transport binders because the net tunnel for substrates was distinct from the product exit tunnel (α-tocopherol acetate). However, compared to results described in the literature using CALB as a biocatalyst to obtain α-tocopherol acetate, it was reinforced as well as other biocatalysts that were used (LBC and LCR) did not obtain promising results. Therefore, the *in silico* analysis of biocatalytic reactions has shown significant results in analyses before *in situ* reactions. It is important to point out that when performing the *in silico* analysis, it demonstrates optimization of the execution time in bench experiments, as it performs a prediction of the submitted biocatalytic behavior.

## Data Availability

The original contributions presented in the study are included in the article/Supplementary Material; further inquiries can be directed to the corresponding author.

## Abbreviations

CALB, *Candida antarctica* B lipase; LTL, *Thermomyces lanuginosus* lipase; LPP, porcine pancreas lipase; RML, *Rhizomucor miehei* lipase; RNL, *Rhizopus niveus* lipase; PBLL, *Photobacterium lipolyticum* lipase; HGL, human gastric lipase; LBC, *Burkholderia cepacia* lipase; LCR, *Candida rugosa* lipase; PDB, Protein Data Bank; SMILES, simplified molecular input line entry system.
