# Supplementary material for: In Silico Evaluation of Enzymatic Tunnels in the Biotransformation of α-Tocopherol Esters
Source: Front Bioeng Biotechnol. 2022 Jan 21;9:805059. doi: 10.3389/fbioe.2021.805059 (PMC8814584; doi:10.3389/fbioe.2021.805059)
Supplement: Supplementary file 1 [file DataSheet1.docx]

Supplementary Material

Supplementary Table 1. Assessment of alpha tocopherol transport in identified LPP tunnels

| **Tunnel ID** | **Ligand** | **E_Bound_**  **[kcal/mol]** | **E_Max_**  **[kcal/mol]** | **E_Surface_**  **[kcal/mol]** | **E_a_**  **[kcal/mol]** | **ΔEBS**  **[kcal/mol]** |
| --- | --- | --- | --- | --- | --- | --- |
| 1 | alpha tocopherol | -4.9 | -4.3 | -5.4 | 1.1 | 0.5 |
| 2 | alpha tocopherol | -5.6 | -4.5 | -4.5 | 0.0 | -1.1 |
| 3 | alpha tocopherol | -5.5 | -4.4 | -4.4 | 0.0 | -1.1 |
| 4 | alpha tocopherol | -5.4 | -4.8 | -4.8 | 0.0 | -0.6 |
| 5 | alpha tocopherol | - | - | - | - | - |
| 6 | alpha tocopherol | -6.2 | -4.1 | -6.0 | 1.9 | -0.2 |
| 7 | alpha tocopherol | -5.0 | -4.1 | -4.3 | 0.2 | -0.7 |
| 8 | alpha tocopherol | -6.1 | -4.1 | -4.2 | 0.1 | -1.9 |
| 9 | alpha tocopherol | -6.3 | -4.2 | -4.7 | 0.5 | -1.6 |
| 10 | alpha tocopherol | -5.8 | -3.7 | -4.3 | 0.6 | -1.5 |
| 11 | alpha tocopherol | -5.4 | 3.8 | -5.7 | 9.5 | 0.3 |
| 12 | alpha tocopherol | -6.0 | -0.6 | -4.8 | 4.2 | -.1.2 |
| 13 | alpha tocopherol | -5.9 | -4.0 | -4.8 | 0.8 | -1.1 |
| 14 | alpha tocopherol | -5.6 | 0.8 | -4.1 | 4.9 | -1.5 |
| 15 | alpha tocopherol | -5.4 | -2.1 | -4.4 | 2.3 | -1.0 |
| 16 | alpha tocopherol | -5.3 | -0.9 | -4.4 | 3.5 | -0.9 |
| 17 | alpha tocopherol | -5.2 | -2.8 | -3.9 | 1.1 | -1.3 |
| 18 | alpha tocopherol | -5.8 | -3.6 | -3.8 | 0.2 | -2.0 |
| 19 | alpha tocopherol | -5.7 | -4.0 | -4.4 | 0.4 | -1.3 |
| 20 | alpha tocopherol | -5.8 | -3.8 | -4.4 | 0.6 | -1.4 |
| 21 | alpha tocopherol | -5.8 | 15.9 | -5.0 | 20.9 | -0.8 |
| 22 | alpha tocopherol | -5.3 | -3.5 | -4.4 | 0.9 | -0.9 |
| 23 | alpha tocopherol | -5.6 | -3.7 | -3.9 | 0.2 | -1.7 |
| 24 | alpha tocopherol | -6.0 | 45.4 | -2.2 | 47.6 | -3.8 |
| 25 | alpha tocopherol | -5.7 | 39.3 | -5.0 | 44.3 | -0.7 |
| 26 | alpha tocopherol | -5.9 | -3.6 | -3.8 | 0.2 | 2.1 |
| 27 | alpha tocopherol | -5.9 | -3.9 | -4.6 | 0.7 | -1.3 |
| 28 | alpha tocopherol | -5.6 | -2.7 | -4.2 | 1.5 | -1.4 |
| 29 | alpha tocopherol | -5.7 | -3.3 | -4.8 | 1.5 | -0.9 |
| 30 | alpha tocopherol | -5.6 | 39.3 | -5.2 | 44.5 | -0.4 |
| 31 | alpha tocopherol | -6.3 | -3.8 | -4.5 | 0.7 | -1.8 |
| 32 | alpha tocopherol | -5.6 | -3.4 | -4.1 | 0.7 | -1.5 |
| 33 | alpha tocopherol | -5.6 | -2.1 | -4.4 | 2.3 | -2.2 |
| 34 | alpha tocopherol | -5.8 | -3.6 | -4.5 | 0.9 | -1.3 |
| 35 | alpha tocopherol | - | - | - | - | - |
| 36 | alpha tocopherol | -5.6 | -1.6 | -3.5 | 1.9 | -2.1 |
| 37 | alpha tocopherol | -5.8 | -3.6 | -4.9 | 1.3 | -0.9 |
| 38 | alpha tocopherol | - | - | - | - | - |
| 39 | alpha tocopherol | -5.8 | 44.9 | -3.6 | 48.5 | -2.2 |

Supplementary Table 2. Assessment of transport of acetylating agents in identified PPL tunnels.

| **Tunnel ID** | **Ligand** | **E_Bound_**  **[kcal/mol]** | **E_Max_**  **[kcal/mol]** | **E_Surface_**  **[kcal/mol]** | **E_a_**  **[kcal/mol]** | **ΔEBS**  **[kcal/mol]** |
| --- | --- | --- | --- | --- | --- | --- |
| 1 | vinyl acetate | -2.1 | -2 | -2.1 | 0.1 | 0 |
|  | vinyl hydrogen succinate | -3.3 | -2.8 | 2.8 | 0.0 | -0.5 |
|  | vinyl ferulate | -4.3 | -3.9 | -3.9 | 0.0 | -0.4 |
|  | vinyl nicotinate | -3.7 | -3.1 | -3.1 | 0.0 | -0.6 |
| 2 | vinyl acetate | -2.1 | -2 | -2.8 | 0.8 | 0.7 |
|  | vinyl hydrogen succinate | -3.3 | -3 | -3.3 | 0.3 | 0.0 |
|  | vinyl ferulate | -4.1 | -4 | -4.4 | 0.4 | 0.3 |
|  | vinyl nicotinate | -3.4 | -3.2 | -3.9 | 0.7 | 0.5 |
| 3 | vinyl acetate | -2.2 | -2.0 | -2.2 | 0.2 | 0 |
|  | vinyl hydrogen succinate | -3.2 | -2.8 | -2.8 | 0 | -0.4 |
|  | vinyl ferulate | -4.0 | -3.6 | -3.9 | 0.3 | -0.1 |
|  | vinyl nicotinate | -3.4 | -3.1 | -3.2 | 0.1 | -0.2 |
| 4 | vinyl acetate | -2.3 | -2.1 | -2.6 | 0.5 | 0.3 |
|  | vinyl hydrogen succinate | -3.4 | -3 | -3.7 | 0.7 | 0.3 |
|  | vinyl ferulate | -4.5 | -4.2 | -4.2 | 0 | -0.3 |
|  | vinyl nicotinate | -3.6 | -3.4 | -3.9 | 0.5 | 0.3 |
| 5 | vinyl acetate | - | - | - | - | - |
|  | vinyl hydrogen succinate | -3.1 | -2.8 | -3.0 | 0.2 | -0.1 |
|  | vinyl ferulate | - | - | - | - | - |
|  | vinyl nicotinate | - | - | - | - | - |
| 6 | vinyl acetate | -2.2 | -2.0 | -2.3 | 0.3 | 0.1 |
|  | vinyl hydrogen succinate | -3.1 | -2.8 | -3 | 0.2 | -0.1 |
|  | vinyl ferulate | -4 | -3.9 | -4.3 | 0.4 | 0.3 |
|  | vinyl nicotinate | -3.6 | -3.2 | -3.4 | 0.2 | -0.2 |
| 7 | vinyl acetate | -2.3 | -1.6 | -1.7 | 0.1 | -0.6 |
|  | vinyl hydrogen succinate | -3.4 | -2.2 | -2.2 | 0.0 | -1.2 |
|  | vinyl ferulate | -4.3 | -3.0 | -3.1 | 0.1 | -1.2 |
|  | vinyl nicotinate | -3.6 | -2.4 | -2.5 | 0.1 | -1-1 |
| 8 | vinyl acetate | -2.2 | -1.5 | -1.6 | 0.1 | -0.6 |
|  | vinyl hydrogen succinate | -3.2 | -2.0 | -2.2 | 0.2 | -1.0 |
|  | vinyl ferulate | -4.6 | -2.7 | -2.7 | 0.0 | -1.9 |
|  | vinyl nicotinate | -3.6 | -2.1 | -2.3 | 0.2 | -1.3 |
| 9 | vinyl acetate | -2.2 | -2.1 | -2.4 | 0.3 | 0.2 |
|  | vinyl hydrogen succinate | -3.2 | -2.9 | -3.1 | 0.2 | -0.1 |
|  | vinyl ferulate | -4.2 | -3.9 | -4.3 | 0.4 | 0.1 |
|  | vinyl nicotinate | -3.7 | -3.2 | -3.8 | 0.6 | 0.1 |
| 10 | vinyl acetate | -2.2 | -2.0 | -2.1 | 0.1 | -0.1 |
|  | vinyl hydrogen succinate | -3.3 | -2.4 | -2.8 | 0.4 | -0.5 |
|  | vinyl ferulate | -4.4 | -2.9 | -2.9 | 0.0 | -1.5 |
|  | vinyl nicotinate | -3.5 | -2.3 | -2.6 | 0.3 | -0.9 |
| 11 | vinyl acetate | -2.3 | -1.4 | -2.5 | 1.1 | 0.2 |
|  | vinyl hydrogen succinate | -3.2 | -2.0 | -3.1 | 1.1 | -0.1 |
|  | vinyl ferulate | -4.8 | -0.8 | -4 | 3.2 | -0.8 |
|  | vinyl nicotinate | -3.4 | 0.4 | -3.6 | 4.0 | 0.2 |
| 12 | vinyl acetate | -2.4 | -2.1 | -2.2 | 0.1 | -0.2 |
|  | vinyl hydrogen succinate | -3.2 | -2.9 | -3.1 | 0.2 | -0.1 |
|  | vinyl ferulate | -4.5 | -4.0 | -4.3 | 0.3 | -0.2 |
|  | vinyl nicotinate | -3.5 | -3.4 | -3.6 | 0.2 | 0.1 |
| 13 | vinyl acetate | -2.3 | -2.0 | -2.6 | 0.6 | 0.3 |
|  | vinyl hydrogen succinate | -3.1 | -2.8 | -3.2 | 0.4 | 0.1 |
|  | vinyl ferulate | -4.2 | -3.7 | -4.0 | 0.3 | -0.2 |
|  | vinyl nicotinate | -3.6 | -3.1 | -4.1 | 1.0 | 0.5 |
| 14 | vinyl acetate | -2.4 | -1.9 | -1.9 | 0.0 | -0.5 |
|  | vinyl hydrogen succinate | -3.2 | -2.7 | -2.7 | 0.0 | -0.5 |
|  | vinyl ferulate | -4.3 | -3.7 | -4.2 | 0.5 | -0.1 |
|  | vinyl nicotinate | -3.5 | -3.0 | -3.2 | 0.2 | -0.3 |
| 15 | vinyl acetate | -2.2 | -1.9 | -2 | 0.1 | -0.2 |
|  | vinyl hydrogen succinate | -3.2 | -2.7 | -3.1 | 0.4 | -0.1 |
|  | vinyl ferulate | -4.3 | -3.0 | -4.4 | 1.4 | 0.1 |
|  | vinyl nicotinate | -3.6 | -2.3 | -3.1 | 0.8 | -0.5 |
| 16 | vinyl acetate | -2.2 | -0.5 | -2.6 | 2.1 | 0.4 |
|  | vinyl hydrogen succinate | -3.1 | -0.1 | -3.6 | 3.5 | 0.5 |
|  | vinyl ferulate | -4.0 | -0.4 | -4.4 | 4.0 | 0.4 |
|  | vinyl nicotinate | -3.4 | 1.0 | -4.1 | 5.1 | 0.7 |
| 17 | vinyl acetate | -2.3 | -1.7 | -1.7 | 0.0 | -0.6 |
|  | vinyl hydrogen succinate | -3.2 | -2.3 | -2.4 | 0.1 | -0.8 |
|  | vinyl ferulate | -4.0 | -2.8 | -2.8 | 0.0 | -1.2 |
|  | vinyl nicotinate | -3.4 | -1.8 | -1.8 | 0.0 | -1.6 |
| 18 | vinyl acetate | -2.2 | -1.7 | -1.9 | 0.2 | -0.3 |
|  | vinyl hydrogen succinate | -3.2 | -2.2 | -2.3 | 0.1 | -0.9 |
|  | vinyl ferulate | -4.3 | -2.8 | -2.8 | 0.0 | -1.5 |
|  | vinyl nicotinate | -3.5 | -2.4 | -2.4 | 0.0 | -1.1 |
| 19 | vinyl acetate | -2.2 | -1.8 | -2.2 | 0.4 | 0.0 |
|  | vinyl hydrogen succinate | -3.1 | -2.3 | -3.2 | 0.9 | 0.1 |
|  | vinyl ferulate | -4.5 | -3.1 | -4.5 | 1.4 | 0.0 |
|  | vinyl nicotinate | -3.5 | -2.6 | -3.5 | 0.9 | 0.0 |
| 20 | vinyl acetate | -2.3 | -1.8 | -2.3 | 0.5 | 0.0 |
|  | vinyl hydrogen succinate | -3.0 | -2.3 | -3 | 0.7 | 0.0 |
|  | vinyl ferulate | -4.5 | -3.1 | -4.2 | 1.1 | -0.3 |
|  | vinyl nicotinate | -3.6 | -2.6 | -3.6 | 1.0 | 0 |
| 21 | vinyl acetate | -2.1 | 4.4 | -1.9 | 6.3 | 0.2 |
|  | vinyl hydrogen succinate | -3.2 | 3.1 | -3 | 6.1 | -0.2 |
|  | vinyl ferulate | -4.4 | 6.3 | -3.5 | 9.8 | -0.9 |
|  | vinyl nicotinate | -3.6 | 8.1 | -2.8 | 10.9 | -0.8 |
| 22 | vinyl acetate | -2.3 | -1.6 | -1.6 | 0.0 | -0.7 |
|  | vinyl hydrogen succinate | -3.3 | -2.2 | -2.2 | 0.0 | -1.1 |
|  | vinyl ferulate | -4.1 | -3.2 | -3.3 | 0.1 | -0.8 |
|  | vinyl nicotinate | -3.7 | -2.7 | -2.8 | 0.1 | -0.9 |
| 23 | vinyl acetate | -2.2 | -1.7 | -1.7 | 0.0 | -0.5 |
|  | vinyl hydrogen succinate | -3.2 | -2.4 | -2.4 | 0.0 | -0.8 |
|  | vinyl ferulate | -4.5 | -3.2 | -3.2 | 0.0 | -1.3 |
|  | vinyl nicotinate | -3.6 | -2.6 | -2.9 | 0.3 | -0.7 |
| 24 | vinyl acetate | -2.4 | 11 | -1.2 | 12.2 | -1.2 |
|  | vinyl hydrogen succinate | -3 | 13.5 | -1.7 | 15.2 | -1.3 |
|  | vinyl ferulate | -4.4 | 27.5 | -0.9 | 28.4 | -3.5 |
|  | vinyl nicotinate | -3.6 | 23.1 | 1.1 | 22.0 | -4.7 |
| 25 | vinyl acetate | -2.2 | 13.9 | -2.5 | 16.4 | 0.3 |
|  | vinyl hydrogen succinate | -3 | 12.4 | -3.3 | 15.7 | 0.3 |
|  | vinyl ferulate | -4.4 | 21.5 | -5.1 | 26.6 | 0.7 |
|  | vinyl nicotinate | -3.5 | 24.9 | -3.9 | 28.8 | 0.4 |
| 26 | vinyl acetate | -2.3 | -1.5 | -1.5 | 0.0 | -0.8 |
|  | vinyl hydrogen succinate | -2.9 | -2.2 | -2.3 | 0.1 | -0.6 |
|  | vinyl ferulate | -4.3 | -3 | -3 | 0.0 | -1.3 |
|  | vinyl nicotinate | -3.6 | -2.4 | -2.6 | 0.2 | -1 |
| 27 | vinyl acetate | -2.3 | -1.8 | -1.8 | 0.0 | -0.5 |
|  | vinyl hydrogen succinate | -3.1 | -2.4 | -2.7 | 0.3 | -0.4 |
|  | vinyl ferulate | -4.3 | -3.1 | -3.9 | 0.8 | -0.4 |
|  | vinyl nicotinate | -3.7 | -2.7 | -2.9 | 0.2 | -0.8 |
| 28 | vinyl acetate | -2.3 | -1.8 | -2.4 | 0.6 | 0.1 |
|  | vinyl hydrogen succinate | -2.9 | -2.6 | -3.3 | 0.7 | 0.4 |
|  | vinyl ferulate | -4.6 | -3.3 | -3.3 | 0.0 | -1.3 |
|  | vinyl nicotinate | -3.5 | -2.8 | -3.6 | 0.8 | 0.1 |
| 29 | vinyl acetate | -2.3 | -1.9 | -2.3 | 0.4 | 0.0 |
|  | vinyl hydrogen succinate | -3.2 | -2.5 | -2.9 | 0.4 | -0.3 |
|  | vinyl ferulate | -4.6 | -3.2 | -4.4 | 1.2 | -0.2 |
|  | vinyl nicotinate | -3.6 | -2.8 | -3.4 | 0.6 | -0.2 |
| 30 | vinyl acetate | -2.2 | 14.1 | -2.2 | 16.3 | 0.0 |
|  | vinyl hydrogen succinate | -3.2 | 12.4 | -3.3 | 15.7 | 0.1 |
|  | vinyl ferulate | -4.1 | 21 | -4.5 | 25.5 | 0.4 |
|  | vinyl nicotinate | -3.5 | 25.2 | -3.4 | 28.6 | -0.1 |
| 31 | vinyl acetate | -2.2 | -1.7 | -2.2 | 0.5 | 0.0 |
|  | vinyl hydrogen succinate | -3.1 | -2.3 | -3.4 | 1.1 | 0.3 |
|  | vinyl ferulate | -4.5 | -3.1 | -4.0 | 0.9 | -0.5 |
|  | vinyl nicotinate | -3.6 | -2.7 | -3.3 | 0.6 | -0.3 |
| 32 | vinyl acetate | -2.2 | -1.5 | -1.7 | 0.2 | -0.5 |
|  | vinyl hydrogen succinate | -3.3 | -2.2 | -2.3 | 0.1 | -1.0 |
|  | vinyl ferulate | -4.5 | -3.0 | -3.6 | 0.6 | -0.9 |
|  | vinyl nicotinate | -3.6 | -2.4 | -2.7 | 0.3 | -0.9 |
| 33 | vinyl acetate | -2.3 | -1.8 | -2.0 | 0.2 | -0.3 |
|  | vinyl hydrogen succinate | -3.1 | -2.4 | -2.5 | 0.1 | -0.6 |
|  | vinyl ferulate | -4.4 | -3.0 | -3.3 | 0.3 | -1.1 |
|  | vinyl nicotinate | -3.7 | -2.4 | -2.7 | 0.3 | -1.0 |
| 34 | vinyl acetate | -2.2 | -1.6 | -1.9 | 0.3 | -0.3 |
|  | vinyl hydrogen succinate | -2.9 | -2.3 | -2.6 | 0.3 | -0.3 |
|  | vinyl ferulate | -3.9 | -3.0 | -3.9 | 0.9 | 0 |
|  | vinyl nicotinate | -3.5 | -2.5 | -2.8 | 0.3 | -0.8 |
| 35 | vinyl acetate | -2.3 | 16.9 | -1.2 | 18.1 | -1.1 |
|  | vinyl hydrogen succinate | -3.2 | 17.6 | -0.5 | 18.1 | -2.7 |
|  | vinyl ferulate | -4.4 | 40.7 | 3.3 | 37.4 | -7.7 |
|  | vinyl nicotinate | -3.6 | 34.5 | 4.0 | 30.5 | -7.6 |
| 36 | vinyl acetate | -2.3 | -1.8 | -2.3 | 0.5 | 0.0 |
|  | vinyl hydrogen succinate | -3.2 | -2.4 | -3.2 | 0.8 | 0.0 |
|  | vinyl ferulate | -4.1 | -2.8 | -3.7 | 0.9 | -0.4 |
|  | vinyl nicotinate | -3.6 | -2.5 | -2.8 | 0.3 | -0.8 |
| 37 | vinyl acetate | -2.3 | -1.9 | -2.7 | 0.8 | 0.4 |
|  | vinyl hydrogen succinate | -3.2 | -2.4 | -3.4 | 1.0 | 0.2 |
|  | vinyl ferulate | -4.4 | -3.0 | -4.1 | 1.1 | -0.3 |
|  | vinyl nicotinate | -3.6 | -2.7 | -3.8 | 1.1 | 0.2 |
| 38 | vinyl acetate | -2.3 | 17.2 | -2.6 | 19.8 | 0.3 |
|  | vinyl hydrogen succinate | -3.1 | 17.9 | -4.0 | 21.9 | 0.9 |
|  | vinyl ferulate | -4.2 | 39.3 | -5.6 | 44.9 | 1.4 |
|  | vinyl nicotinate | -3.5 | 34.2 | -4.3 | 38.5 | 0.8 |
| 39 | vinyl acetate | -2.1 | 15.8 | -2.5 | 18.3 | 0.4 |
|  | vinyl hydrogen succinate | -3.0 | 16.3 | -3.4 | 19.7 | 0.4 |
|  | vinyl ferulate | -4.4 | 28.2 | -3.8 | 32 | -0.6 |
|  | vinyl nicotinate | -3.6 | 28.2 | -3.6 | 31.8 | 0.0 |

Supplementary Table 3. Assessment of product transport in LPP identified tunnels

| **Tunnel ID** | **Ligand** | **E_Bound_**  **[kcal/mol]** | **E_Max_**  **[kcal/mol]** | **E_Surface_**  **[kcal/mol]** | **E_a_**  **[kcal/mol]** | **ΔEBS**  **[kcal/mol]** |
| --- | --- | --- | --- | --- | --- | --- |
| 1 | alpha-Tocopherol acetate | -4.4 | -4.4 | -5.7 | 0.0 | 1.3 |
|  | alpha-Tocopherol succinate | -4.8 | -4.4 | -5.9 | 0.4 | 1.1 |
|  | alpha-Tocopherol ferrulate | -4.8 | -4.8 | -6.2 | 0.0 | 1.4 |
|  | alpha-Tocopherol nicotinate | -5.1 | -4.5 | -6.7 | 0.6 | 1.6 |
| 2 | alpha-Tocopherol acetate | -4.5 | -4.1 | -5.4 | 0.4 | 0.9 |
|  | alpha-Tocopherol succinate | -4.7 | -4.3 | -5.4 | 0.4 | 0.7 |
|  | alpha-Tocopherol ferrulate | -5.0 | -4.6 | -7.9 | 0.4 | 2.9 |
|  | alpha-Tocopherol nicotinate | -4.9 | -4.5 | -5.9 | 0.4 | 1.0 |
| 3 | alpha-Tocopherol acetate | -4.6 | -4.1 | -5.3 | 0.5 | 0.7 |
|  | alpha-Tocopherol succinate | -4.9 | -4.5 | -5.9 | 0.4 | 1.0 |
|  | alpha-Tocopherol ferrulate | -5.0 | -4.7 | -6.4 | 0.3 | 1.4 |
|  | alpha-Tocopherol nicotinate | -5.7 | -5 | -5.8 | 0.7 | 0.1 |
| 4 | alpha-Tocopherol acetate* | -4.6 | -4.6 | -6.2 | 0.0 | 1.6 |
|  | alpha-Tocopherol succinate* | -4.8 | -4.8 | -5.7 | 0.0 | 0.9 |
|  | alpha-Tocopherol ferrulate | -4.8 | -4.7 | -6.5 | 0.1 | 1.7 |
|  | alpha-Tocopherol nicotinate | -4.8 | -4.8 | -5.4 | 0.0 | 0.6 |
| 5 | alpha-Tocopherol acetate* | - | - | - | - | - |
|  | alpha-Tocopherol succinate* | - | - | - | - | - |
|  | alpha-Tocopherol ferrulate | - | - | - | - | - |
|  | alpha-Tocopherol nicotinate | - | - | - | - | - |
| 6 | alpha-Tocopherol acetate* | -5.5 | -4.6 | -5.3 | 0.9 | -0.2 |
|  | alpha-Tocopherol succinate* | -5.1 | -4.6 | -5.9 | 0.5 | 0.8 |
|  | alpha-Tocopherol ferrulate | -5.9 | -3.8 | -6.3 | 2.1 | 0.4 |
|  | alpha-Tocopherol nicotinate | -5.6 | -5.0 | -5.9 | 0.6 | 0.3 |
| 7 | alpha-Tocopherol acetate* | -3.9 | -3.8 | -5.2 | 0.1 | 1.3 |
|  | alpha-Tocopherol succinate* | -4.4 | -4.3 | -5.6 | 0.1 | 1.2 |
|  | alpha-Tocopherol ferrulate | -5.4 | -4.5 | -6.1 | 0.9 | 0.7 |
|  | alpha-Tocopherol nicotinate | -5.6 | -4.3 | -6.0 | 1.3 | 0.4 |
| 8 | alpha-Tocopherol acetate* | -3.9 | -3.4 | -5.5 | 0.5 | 1.6 |
|  | alpha-Tocopherol succinate* | -3.8 | -3.6 | -5.5 | 0.2 | 1.7 |
|  | alpha-Tocopherol ferrulate | -3.8 | -3.7 | -5.9 | 0.1 | 2.1 |
|  | alpha-Tocopherol nicotinate | -4.6 | -4 | -6 | 0.6 | 1.4 |
| 9 | alpha-Tocopherol acetate* | -4.6 | -4.5 | -4.5 | 0.1 | -0.1 |
|  | alpha-Tocopherol succinate* | -4.8 | -4.5 | -5.6 | 0.3 | 0.8 |
|  | alpha-Tocopherol ferrulate | -5.1 | -4.2 | -6.5 | 0.9 | 1.4 |
|  | alpha-Tocopherol nicotinate | -5.2 | -4.7 | -6.3 | 0.5 | 1.1 |
| 10 | alpha-Tocopherol acetate* | -4.2 | -3.7 | -5.3 | 0.5 | 1.1 |
|  | alpha-Tocopherol succinate* | -4.5 | -4.0 | -5.4 | 0.5 | 0.9 |
|  | alpha-Tocopherol ferrulate | -4.2 | -4.0 | -5.6 | 0.2 | 1.4 |
|  | alpha-Tocopherol nicotinate | -4.8 | -4.3 | -5.6 | 0.5 | 0.8 |
| 11 | alpha-Tocopherol acetate* | -4.5 | 0.2 | -5.2 | 4.7 | 0.7 |
|  | alpha-Tocopherol succinate* | -5 | 2.1 | -6.2 | 7.1 | 1.2 |
|  | alpha-Tocopherol ferrulate | -5.2 | 5.0 | -6.6 | 10.2 | 1.4 |
|  | alpha-Tocopherol nicotinate | -5.5 | 2.1 | -5.6 | 7.6 | 0.1 |
| 12 | alpha-Tocopherol acetate* | -5.0 | -2.3 | -5.1 | 2.7 | 0.1 |
|  | alpha-Tocopherol succinate* | -4.9 | -0.9 | -6.1 | 4.0 | 1.2 |
|  | alpha-Tocopherol ferrulate | -5.0 | 0.1 | -6.7 | 5.1 | 1.7 |
|  | alpha-Tocopherol nicotinate | -4.6 | -1.9 | -5.7 | 2.7 | 1.1 |
| 13 | alpha-Tocopherol acetate* | -4.5 | -4.1 | -5.5 | 0.4 | 1.0 |
|  | alpha-Tocopherol succinate* | -5.4 | -4.3 | -5.6 | 1.1 | 0.2 |
|  | alpha-Tocopherol ferrulate | -5.0 | -3.9 | -6.2 | 1.1 | 1.2 |
|  | alpha-Tocopherol nicotinate | -5.6 | -4.7 | -6.3 | 0.9 | 0.7 |
| 14 | alpha-Tocopherol acetate* | -4.5 | -2.3 | -5.4 | 2.2 | 0.9 |
|  | alpha-Tocopherol succinate* | -5.2 | -1.5 | -5.9 | 3.7 | 0.7 |
|  | alpha-Tocopherol ferrulate | -4.6 | 0.3 | -5.9 | 4.9 | 1.3 |
|  | alpha-Tocopherol nicotinate | -4.6 | -1.8 | -6.1 | 2.8 | 1.5 |
| 15 | alpha-Tocopherol acetate* | -4.0 | -3.1 | -5.6 | 0.9 | 1.6 |
|  | alpha-Tocopherol succinate* | -4.6 | -3.6 | -5.6 | 1.0 | 1.0 |
|  | alpha-Tocopherol ferrulate | -4.6 | -3.8 | -5.8 | 0.8 | 1.2 |
|  | alpha-Tocopherol nicotinate | -4.7 | -4.1 | -5.7 | 0.6 | 1.0 |
| 16 | alpha-Tocopherol acetate* | -4.6 | -2.1 | -5.3 | 2.5 | 0.7 |
|  | alpha-Tocopherol succinate* | -5.0 | -2.1 | -5.7 | 2.9 | 0.7 |
|  | alpha-Tocopherol ferrulate | -5.5 | -0.4 | -6.1 | 5.1 | 0.6 |
|  | alpha-Tocopherol nicotinate | -4.8 | -2.6 | -5.9 | 2.2 | 1.1 |
| 17 | alpha-Tocopherol acetate* | -2.5 | -1.7 | -5.3 | 0.8 | 2.8 |
|  | alpha-Tocopherol succinate* | -2.0 | -1.7 | -5.6 | 0.3 | 3.6 |
|  | alpha-Tocopherol ferrulate | 3.2 | 3.2 | -5.8 | 0.0 | 9.0 |
|  | alpha-Tocopherol nicotinate | -1.9 | -1.9 | -5.4 | 0.0 | 3.5 |
| 18 | alpha-Tocopherol acetate* | -4.4 | -3.8 | -5.4 | 0.6 | 1.0 |
|  | alpha-Tocopherol succinate* | -4.0 | -3.7 | -5.3 | 0.3 | 1.3 |
|  | alpha-Tocopherol ferrulate | -4.4 | -4.1 | -6.2 | 0.3 | 1.8 |
|  | alpha-Tocopherol nicotinate | -4.2 | -4.2 | -6.5 | 0.0 | 2.3 |
| 19 | alpha-Tocopherol acetate* | -4.2 | -4.2 | -6.0 | 0.0 | 1.8 |
|  | alpha-Tocopherol succinate* | -5.0 | -4.4 | -5.8 | 0.6 | 0.8 |
|  | alpha-Tocopherol ferrulate | -5.0 | -4.1 | -6.0 | 0.9 | 1.0 |
|  | alpha-Tocopherol nicotinate | -6.0 | -4.6 | -5.5 | 1.4 | -0.5 |
| 20 | alpha-Tocopherol acetate* | -4.7 | -4.1 | -5.0 | 0.6 | 0.3 |
|  | alpha-Tocopherol succinate* | -5.8 | -4.2 | -5.7 | 1.6 | -0.1 |
|  | alpha-Tocopherol ferrulate | -6.0 | -4.0 | -6.2 | 2.0 | 0.2 |
|  | alpha-Tocopherol nicotinate | -5.8 | -4.4 | -6.3 | 1.4 | 0.5 |
| 21 | alpha-Tocopherol acetate* | -4.3 | 10.2 | -6.2 | 14.5 | 1.9 |
|  | alpha-Tocopherol succinate* | -4.6 | 8.9 | -5.1 | 13.5 | 0.5 |
|  | alpha-Tocopherol ferrulate | -4.3 | 8.4 | -6.5 | 12.7 | 2.2 |
|  | alpha-Tocopherol nicotinate | -5 | 9.4 | -5.7 | 14.4 | 0.7 |
| 22 | alpha-Tocopherol acetate* | -3.7 | -3.7 | -5.1 | 0.0 | 1.4 |
|  | alpha-Tocopherol succinate* | -4.5 | -4.1 | -6.1 | 0.4 | 1.6 |
|  | alpha-Tocopherol ferrulate | -4.3 | -4.1 | -5.7 | 0.2 | 1.4 |
|  | alpha-Tocopherol nicotinate | -4.9 | -4.5 | -6.1 | 0.4 | 1.2 |
| 23 | alpha-Tocopherol acetate* | -3.2 | -3.2 | -5.1 | 0.0 | 1.9 |
|  | alpha-Tocopherol succinate* | -3.9 | -3.6 | -5.8 | 0.3 | 1.9 |
|  | alpha-Tocopherol ferrulate | -4.4 | -3.8 | -6.6 | 0.6 | 2.2 |
|  | alpha-Tocopherol nicotinate | -3.8 | -3.5 | -5.1 | 0.3 | 1.3 |
| 24 | alpha-Tocopherol acetate* | -3.7 | 37.1 | -5.1 | 40.8 | 1.4 |
|  | alpha-Tocopherol succinate* | -4.1 | 36.5 | -5.8 | 40.6 | 1.7 |
|  | alpha-Tocopherol ferrulate | - | - | - | - | - |
|  | alpha-Tocopherol nicotinate | -3.9 | 39.0 | -6.3 | 42.9 | 2.4 |
| 25 | alpha-Tocopherol acetate* | -4.7 | 28.5 | -5.9 | 33.2 | 1.2 |
|  | alpha-Tocopherol succinate* | -5.7 | 30.4 | -5.5 | 36.1 | -0.2 |
|  | alpha-Tocopherol ferrulate | - | - | - | - | - |
|  | alpha-Tocopherol nicotinate | -5.3 | 35.4 | -5.9 | 40.7 | 0.6 |
| 26 | alpha-Tocopherol acetate* | -3.6 | -3.4 | -5.5 | 0.2 | 1.9 |
|  | alpha-Tocopherol succinate* | -4.0 | -4.0 | -5.5 | 0.0 | 1.5 |
|  | alpha-Tocopherol ferrulate | -4.5 | -3.8 | -6.3 | 0.7 | 1.8 |
|  | alpha-Tocopherol nicotinate | -4.5 | -4.2 | -5.6 | 0.3 | 1.1 |
| 27 | alpha-Tocopherol acetate* | -4.4 | -4.1 | -5.4 | 0.3 | 1.0 |
|  | alpha-Tocopherol succinate* | -4.0 | -4.0 | -5.9 | 0.0 | 1.9 |
|  | alpha-Tocopherol ferrulate | -4.1 | -4.1 | -6.7 | 0.0 | 2.6 |
|  | alpha-Tocopherol nicotinate | -5.2 | -4.6 | -5.9 | 0.6 | 0.7 |
| 28 | alpha-Tocopherol acetate* | -3.7 | -2.9 | -4.9 | 0.8 | 1.2 |
|  | alpha-Tocopherol succinate* | -3.6 | -3.5 | -6.0 | 0.1 | 2.4 |
|  | alpha-Tocopherol ferrulate | -3.9 | -3.7 | -5.2 | 0.2 | 1.3 |
|  | alpha-Tocopherol nicotinate | -4.0 | -3.6 | -5.9 | 0.4 | 1.9 |
| 29 | alpha-Tocopherol acetate* | -5.1 | -3.9 | -5.5 | 1.2 | 0.4 |
|  | alpha-Tocopherol succinate* | -5 | -4.2 | -6.1 | 0.8 | 1.1 |
|  | alpha-Tocopherol ferrulate | -5.8 | -4.1 | -5.4 | 1.7 | -0.4 |
|  | alpha-Tocopherol nicotinate | -6.1 | -4.8 | -6.2 | 1.3 | 0.1 |
| 30 | alpha-Tocopherol acetate* | -4.4 | 28.4 | -5.2 | 32.8 | 0.8 |
|  | alpha-Tocopherol succinate* | -4.1 | 30.3 | -5.1 | 34.4 | 1.0 |
|  | alpha-Tocopherol ferrulate | - | - | - | - | - |
|  | alpha-Tocopherol nicotinate | -4.6 | 35.2 | -5.4 | 39.8 | 0.8 |
| 31 | alpha-Tocopherol acetate* | -5.7 | -4.2 | -5.1 | 1.5 | -0.6 |
|  | alpha-Tocopherol succinate* | -6.3 | -4.5 | -5.9 | 1.8 | -0.4 |
|  | alpha-Tocopherol ferrulate | 6.3 | -4.3 | -6.7 | 2.0 | 0.4 |
|  | alpha-Tocopherol nicotinate | -6.3 | -4.5 | -6.1 | 1.8 | -0.2 |
| 32 | alpha-Tocopherol acetate* | -4.0 | -4.0 | -5.8 | 0.0 | 1.8 |
|  | alpha-Tocopherol succinate* | -4.3 | -4.1 | -5.8 | 0.2 | 1.5 |
|  | alpha-Tocopherol ferrulate | -4.6 | -3.9 | -6.4 | 0.7 | 1.8 |
|  | alpha-Tocopherol nicotinate | -5.0 | -4.6 | -6.0 | 0.4 | 1.0 |
| 33 | alpha-Tocopherol acetate* | -4.5 | -2.6 | -5.5 | 1.9 | 1.0 |
|  | alpha-Tocopherol succinate* | -4.0 | -3.5 | -5.5 | 0.5 | 1.5 |
|  | alpha-Tocopherol ferrulate | -4.7 | -3.9 | -6.1 | 0.8 | 1.4 |
|  | alpha-Tocopherol nicotinate | -4.2 | -3.6 | -6.7 | 0.6 | 2.5 |
| 34 | alpha-Tocopherol acetate* | -4.5 | -4.0 | -5.8 | 0.5 | 1.3 |
|  | alpha-Tocopherol succinate* | -4.4 | -4.0 | -6.1 | 0.4 | 1.7 |
|  | alpha-Tocopherol ferrulate | -4.8 | -3.7 | -6.9 | 1.1 | 2.1 |
|  | alpha-Tocopherol nicotinate | -4.9 | -4.4 | -5.8 | 0.5 | 0.9 |
| 35 | alpha-Tocopherol acetate* | - | - | - | - | - |
|  | alpha-Tocopherol succinate* | - | - | - | - | -- |
|  | alpha-Tocopherol ferrulate | - | - | - | - | - |
|  | alpha-Tocopherol nicotinate | - | - | - | - | - |
| 36 | alpha-Tocopherol acetate* | -3.4 | -1.5 | -5.3 | 1.9 | 1.9 |
|  | alpha-Tocopherol succinate* | -4.5 | -3.0 | -5.6 | 1.5 | 1.1 |
|  | alpha-Tocopherol ferrulate | -4.7 | -3.3 | -6.6 | 1.4 | 1.9 |
|  | alpha-Tocopherol nicotinate | -4.4 | -2.9 | 6.3 | 1.5 | 1.9 |
| 37 | alpha-Tocopherol acetate* | -4.8 | -4.1 | -5.2 | 0.7 | 0.4 |
|  | alpha-Tocopherol succinate* | -5.1 | -4.2 | -5.6 | 0.9 | 0.5 |
|  | alpha-Tocopherol ferrulate | -5.7 | -4.1 | -6.4 | 1.6 | 0.7 |
|  | alpha-Tocopherol nicotinate | -5.9 | -4.7 | -6.4 | 1.2 | 0.5 |
| 38 | alpha-Tocopherol acetate* | - | - | - | - | - |
|  | alpha-Tocopherol succinate* | - | - | - | - | - |
|  | alpha-Tocopherol ferrulate | - | - | - | - | - |
|  | alpha-Tocopherol nicotinate | - | - | - | - | - |
| 39 | alpha-Tocopherol acetate* | - | - | - | - | - |
|  | alpha-Tocopherol succinate* | - | - | - | - | - |
|  | alpha-Tocopherol ferrulate | - | - | - | - | - |
|  | alpha-Tocopherol nicotinate | - | - | - | - | - |

Supplementary Table 4. Assessment of alpha tocopherol transport in identified HGL tunnels

| **Tunnel ID** | **Ligand** | **E_Bound_**  **[kcal/mol]** | **E_Max_**  **[kcal/mol]** | **E_Surface_**  **[kcal/mol]** | **E_a_**  **[kcal/mol]** | **ΔEBS**  **[kcal/mol]** |
| --- | --- | --- | --- | --- | --- | --- |
| 1 | alpha tocopherol | 7 | 11.4 | 3.2 | 8.2 | 3.8 |
| 2 | alpha tocopherol | 13.5 | 15.3 | 6.7 | 8.6 | 6.8 |
| 3 | alpha tocopherol | 16.0 | 38.4 | 27.5 | 10.9 | -11.5 |
| 4 | alpha tocopherol | - | - | - | - | - |
| 5 | alpha tocopherol | - | - | - | - | - |
| 6 | alpha tocopherol | - | - | - | - | - |
| 7 | alpha tocopherol | - | - | - | - | - |

Supplementary Table 5. Assessment of transport of acetylating agents in tunnels identified in HGL

| **Tunnel ID** | **Ligand** | **E_Bound_**  **[kcal/mol]** | **E_Max_**  **[kcal/mol]** | **E_Surface_**  **[kcal/mol]** | **E_a_**  **[kcal/mol]** | **ΔEBS**  **[kcal/mol]** |
| --- | --- | --- | --- | --- | --- | --- |
| 1 | vinyl acetate | -3.2 | -2.7 | -2.8 | 0.1 | -0.4 |
|  | vinyl hydrogen succinate | -3.8 | -3.3 | -4.0 | 0.7 | 0.2 |
|  | vinyl ferulate | -1.7 | 1.0 | -4.4 | 5.4 | 2.7 |
|  | vinyl nicotinate | -3.6 | 0.2 | -3.4 | 3.6 | -0.2 |
| 2 | vinyl acetate | -2.3 | 0.7 | -2.4 | 3.1 | 0.1 |
|  | vinyl hydrogen succinate | -1.9 | -0.2 | -3.3 | 3.1 | 1.4 |
|  | vinyl ferulate | 3.7 | 4.7 | -2.2 | 6.9 | 5.9 |
|  | vinyl nicotinate | 0.0 | 4.2 | -0.5 | 4.7 | 0.5 |
| 3 | vinyl acetate | 1.4 | 13.3 | -1.5 | 14.8 | 2.9 |
|  | vinyl hydrogen succinate | -0.6 | 13.1 | -1.7 | 14.8 | 1.1 |
|  | vinyl ferulate | 13.8 | 21 | 9.8 | 11.2 | 4.0 |
|  | vinyl nicotinate | 13.7 | 24.5 | 3.6 | 20.9 | 10.1 |
| 4 | vinyl acetate | 1.5 | 16..4 | -2.3 | 18.7 | 3.8 |
|  | vinyl hydrogen succinate | -0.5 | 19 | -1.9 | 20.9 | 1.4 |
|  | vinyl ferulate | 13.3 | 36.2 | 11.1 | 25.1 | 2.2 |
|  | vinyl nicotinate | 13.6 | 29.3 | 3.3 | 26 | 10.3 |
| 5 | vinyl acetate | 1.9 | 16.5 | -2.9 | 19.4 | 4.8 |
|  | vinyl hydrogen succinate | -0.6 | 19.4 | -3 | 22.4 | 2.4 |
|  | vinyl ferulate | 12.8 | 36.7 | -2.6 | 39.3 | 15.4 |
|  | vinyl nicotinate | 13.8 | 30.8 | -2.4 | 33.2 | 16.2 |
| 6 | vinyl acetate | 1.9 | 17.2 | 15.3 | 1.9 | -13.4 |
|  | vinyl hydrogen succinate | -0.9 | 16.7 | 15.3 | 1.4 | -16.2 |
|  | vinyl ferulate | 15.3 | 33.9 | 28.8 | 5.1 | -13.5 |
|  | vinyl nicotinate | 14 | 30.9 | 26.8 | 4.1 | -12.8 |
| 7 | vinyl acetate | 1.5 | 17.2 | -0.4 | 17.6 | 1.9 |
|  | vinyl hydrogen succinate | -0.6 | 20 | -0.7 | 20.7 | 0.1 |
|  | vinyl ferulate | 13 | 39.8 | 11.6 | 28.2 | 1.4 |
|  | vinyl nicotinate | 13.7 | 32.1 | 5.3 | 26.8 | 8.4 |

Supplementary Table 6. Assessment of product transport in tunnels identified in the HGL

| **Tunnel ID** | **Ligand** | **E_Bound_**  **[kcal/mol]** | **E_Max_**  **[kcal/mol]** | **E_Surface_**  **[kcal/mol]** | **E_a_**  **[kcal/mol]** | **ΔEBS**  **[kcal/mol]** |
| --- | --- | --- | --- | --- | --- | --- |
| 1 | alpha-Tocopherol acetate | -4.5 | 6.8 | 6.8 | 11.3 | -11.3 |
|  | alpha-Tocopherol succinate | -4.4 | 7.6 | 6.9 | 12.0 | -11.3 |
|  | alpha-Tocopherol ferrulate | -3.9 | 11.3 | 11.3 | 15.2 | -15.2 |
|  | alpha-Tocopherol nicotinate | -4.1 | 8.7 | 8.7 | 12.8 | -12.8 |
| 2 | alpha-Tocopherol acetate | -3.9 | 10.5 | 7.2 | 14.4 | -11.1 |
|  | alpha-Tocopherol succinate | 0.2 | 12.8 | 8.2 | 12.6 | -8.0 |
|  | alpha-Tocopherol ferrulate | 0.2 | 18.8 | 10.2 | 18.6 | -10 |
|  | alpha-Tocopherol nicotinate | 0.9 | 15.5 | 0.9 | 14.6 | -8.0 |
| 3 | alpha-Tocopherol acetate | -4.4 | 36.8 | 6.9 | 41.2 | -11.3 |
|  | alpha-Tocopherol succinate | -4.0 | 33.8 | 6.4 | 37.8 | -10.4 |
|  | alpha-Tocopherol ferrulate | - | - | - | - | - |
|  | alpha-Tocopherol nicotinate | - | - | - | - | - |
| 4 | alpha-Tocopherol acetate | - | - | - | - | - |
|  | alpha-Tocopherol succinate | - | - | - | - | - |
|  | alpha-Tocopherol ferrulate | - | - | - | - | - |
|  | alpha-Tocopherol nicotinate | - | - | - | - | - |

Supplementary Table 7. Assessment of alpha tocopherol transport in identified RNL tunnels

| **Tunnel ID** | **Ligand** | **E_Bound_**  **[kcal/mol]** | **E_Max_**  **[kcal/mol]** | **E_Surface_**  **[kcal/mol]** | **E_a_**  **[kcal/mol]** | **ΔEBS**  **[kcal/mol]** |
| --- | --- | --- | --- | --- | --- | --- |
| 1 | alpha tocopherol | -4.4 | -3.9 | -4.2 | 0.3 | -0.2 |
| 2 | alpha tocopherol | -4.5 | -4.0 | -4.0 | 0.0 | -0.5 |
| 3 | alpha tocopherol | 2.9 | 15.9 | 9.9 | 6.0 | -7.0 |
| 4 | alpha tocopherol | - | - | - | - | - |

Supplementary Table 8. Assessment of acetylation agent transport in identified RNL tunnels

| **Tunnel ID** | **Ligand** | **E_Bound_**  **[kcal/mol]** | **E_Max_**  **[kcal/mol]** | **E_Surface_**  **[kcal/mol]** | **E_a_**  **[kcal/mol]** | **ΔEBS**  **[kcal/mol]** |
| --- | --- | --- | --- | --- | --- | --- |
| 1 | vinyl acetate | -2.2 | -2.2 | -2.2 | 0.0 | 0.0 |
|  | vinyl hydrogen succinate | -3.0 | -3.0 | -3.1 | 0.1 | 0.1 |
|  | vinyl ferulate | -4.1 | -4.1 | -4.2 | 0.1 | 0.1 |
|  | vinyl nicotinate | -3.3 | -3.3 | -3.5 | 0.2 | 0.2 |
| 2 | vinyl acetate | -2.3 | -2.0 | -2.0 | 0.0 | -0.3 |
|  | vinyl hydrogen succinate | -3.0 | -2.7 | -2.7 | 0.0 | -0.3 |
|  | vinyl ferulate | -3.9 | -3.5 | -3.6 | 0.1 | -0.3 |
|  | vinyl nicotinate | -3.3 | -3.1 | -3.2 | 0.1 | -0.1 |
| 3 | vinyl acetate | -2.3 | 2.1 | -0.9 | 3.0 | -1.4 |
|  | vinyl hydrogen succinate | -3.4 | 1.8 | -1.4 | 3.2 | -2.0 |
|  | vinyl ferulate | -2.9 | 7.0 | 0.6 | 6.4 | -3.5 |
|  | vinyl nicotinate | -1.7 | 5.3 | 3.1 | 2.2 | -4.8 |
| 4 | vinyl acetate | -2.2 | 12.9 | -0.9 | 13.8 | -1.3 |
|  | vinyl hydrogen succinate | -3.2 | 17.1 | -1.0 | 18.1 | -2.2 |
|  | vinyl ferulate | -4.1 | 36.6 | -0.1 | 36.6 | -4.0 |
|  | vinyl nicotinate | -3.3 | 30.1 | 0.9 | 29.2 | -4.2 |

Supplementary Table 9. Assessment of product transport in identified RNL tunnels

| **Tunnel ID** | **Ligand** | **E_Bound_**  **[kcal/mol]** | **E_Max_**  **[kcal/mol]** | **E_Surface_**  **[kcal/mol]** | **E_a_**  **[kcal/mol]** | **ΔEBS**  **[kcal/mol]** |
| --- | --- | --- | --- | --- | --- | --- |
| 1 | alpha-Tocopherol acetate | -4.2 | -4 | -4.5 | 0.2 | 0.3 |
|  | alpha-Tocopherol succinate | -4.1 | -4.1 | -4.3 | 0.0 | 0.2 |
|  | alpha-Tocopherol ferrulate | -4.9 | -4.3 | -5.1 | 0.6 | 0.2 |
|  | alpha-Tocopherol nicotinate | -5.3 | -4.3 | -4.8 | 1.0 | -0.5 |
| 2 | alpha-Tocopherol acetate | -4.2 | -4.1 | -4.4 | 0.1 | 0.2 |
|  | alpha-Tocopherol succinate | -3.7 | -3.7 | -4.6 | 0.0 | 0.9 |
|  | alpha-Tocopherol ferrulate | -4.1 | -4.0 | -4.9 | 0.1 | 0.8 |
|  | alpha-Tocopherol nicotinate | -4.2 | -4.2 | -4.7 | 0.0 | 0.5 |
| 3 | alpha-Tocopherol acetate | -4.4 | 12.8 | -4.7 | 17.2 | 0.3 |
|  | alpha-Tocopherol succinate | -5.2 | 10.7 | -4.5 | 15.9 | -0.7 |
|  | alpha-Tocopherol ferrulate | -5.0 | 15.5 | -3.1 | 20.5 | -1.9 |
|  | alpha-Tocopherol nicotinate | -5.5 | 11.6 | -4.2 | 17.1 | -1.3 |
| 4 | alpha-Tocopherol acetate | - | - | - | - | - |
|  | alpha-Tocopherol succinate | - | - | - | - | - |
|  | alpha-Tocopherol ferrulate | - | - | - | - | - |
|  | alpha-Tocopherol nicotinate | - | - | - | - | - |

Supplementary Table 10. Assessment of alpha tocopherol transport in identified BCL tunnels

| **Tunnel ID** | **Ligand** | **E_Bound_**  **[kcal/mol]** | **E_Max_**  **[kcal/mol]** | **E_Surface_**  **[kcal/mol]** | **E_a_**  **[kcal/mol]** | **ΔEBS**  **[kcal/mol]** |
| --- | --- | --- | --- | --- | --- | --- |
| 1 | alpha tocopherol | -3.4 | 16.9 | -4.6 | 21.5 | 1.2 |
| 2 | alpha tocopherol | 9.3 | 25.4 | 7.7 | 17.7 | 1.6 |
| 3 | alpha tocopherol | 7.5 | 42.3 | 27.3 | 15.0 | -19.8 |
| 4 | alpha tocopherol | -2.8 | 44.4 | -6.2 | 50.6 | 3.4 |

Supplementary Table 11. Assessment of acetylation agent transport in identified BCL tunnels

| **Tunnel ID** | **Ligand** | **E_Bound_**  **[kcal/mol]** | **E_Max_**  **[kcal/mol]** | **E_Surface_**  **[kcal/mol]** | **E_a_**  **[kcal/mol]** | **ΔEBS**  **[kcal/mol]** |
| --- | --- | --- | --- | --- | --- | --- |
| 1 | vinyl acetate | -2.8 | -1.0 | -2.9 | 1.9 | 0.1 |
|  | vinyl hydrogen succinate | -3.8 | -1.4 | -4.0 | 2.6 | 0.2 |
|  | vinyl ferulate | -3.5 | 12.9 | -5.1 | 18.0 | 1.6 |
|  | vinyl nicotinate | -2.5 | 11.7 | -4.1 | 15.8 | 1.6 |
| 2 | vinyl acetate | -2.7 | 8.0 | -3.0 | 11.0 | 0.3 |
|  | vinyl hydrogen succinate | -3.9 | 8.0 | -3.1 | 11.1 | -0.8 |
|  | vinyl ferulate | -1.8 | 16.5 | -3.1 | 19.6 | 1.3 |
|  | vinyl nicotinate | -2.5 | 16.5 | -2.6 | 19.1 | 0.1 |
| 3 | vinyl acetate | -2.7 | 10.9 | 0.0 | 10.9 | -2.7 |
|  | vinyl hydrogen succinate | -3.8 | 12.1 | 0.3 | 11.8 | -4.1 |
|  | vinyl ferulate | -3.3 | 27.2 | 16.4 | 10.8 | -19.7 |
|  | vinyl nicotinate | -2.6 | 24.6 | 10.6 | 14.0 | -13.4 |
| 4 | vinyl acetate | -2.8 | 18.0 | -2.2 | 20.2 | -0.6 |
|  | vinyl hydrogen succinate | -3.9 | 23.3 | -2.7 | 26.0 | -1.2 |
|  | vinyl ferulate | -1.9 | 40.0 | -0.9 | 40.9 | -1.0 |
|  | vinyl nicotinate | -2.4 | 37.3 | 1.1 | 36.2 | -3.5 |

Supplementary Table 12. Assessment of product transport in BCL identified tunnels

| **Tunnel ID** | **Ligand** | **E_Bound_**  **[kcal/mol]** | **E_Max_**  **[kcal/mol]** | **E_Surface_**  **[kcal/mol]** | **E_a_**  **[kcal/mol]** | **ΔEBS**  **[kcal/mol]** |
| --- | --- | --- | --- | --- | --- | --- |
| 1 | alpha-Tocopherol acetate | -2.3 | -0.5 | -7.9 | 1.8 | 5.6 |
|  | alpha-Tocopherol succinate | 10.9 | 10.9 | -7.4 | 0.0 | 18.3 |
|  | alpha-Tocopherol ferrulate | 18.9 | 18.9 | -7.3 | 0.0 | 26.2 |
|  | alpha-Tocopherol nicotinate | 10.9 | 10.9 | -7.6 | 0.0 | 18.5 |
| 2 | alpha-Tocopherol acetate | -4.6 | 22.3 | -3.3 | 26.9 | -1.3 |
|  | alpha-Tocopherol succinate | 0.0 | 20.7 | -3.7 | 20.7 | 3.7 |
|  | alpha-Tocopherol ferrulate | 13.9 | 23.0 | -2.0 | 9.1 | 15.9 |
|  | alpha-Tocopherol nicotinate | -0.9 | 22.8 | -4.1 | 23.7 | 3.2 |
| 3 | alpha-Tocopherol acetate | -4.6 | 20.3 | -4.0 | 24.9 | -0.6 |
|  | alpha-Tocopherol succinate | 1.4 | 36.0 | -1.5 | 34.6 | 2.9 |
|  | alpha-Tocopherol ferrulate | - | - | - | - | - |
|  | alpha-Tocopherol nicotinate | 1.5 | 39.5 | -1.5 | 38.0 | 3.0 |
| 4 | alpha-Tocopherol acetate* | - | - | - | - | - |
|  | alpha-Tocopherol succinate* | - | - | - | - | - |
|  | alpha-Tocopherol ferrulate | - | - | - | - | - |
|  | alpha-Tocopherol nicotinate | - | - | - | - | - |

Supplementary Table 13. Assessment of alpha tocopherol transport in identified PBLL tunnels

| **Tunnel ID** | **Ligand** | **E_Bound_**  **[kcal/mol]** | **E_Max_**  **[kcal/mol]** | **E_Surface_**  **[kcal/mol]** | **E_a_**  **[kcal/mol]** | **ΔEBS**  **[kcal/mol]** |
| --- | --- | --- | --- | --- | --- | --- |
| 1 | alpha tocopherol | -3.5 | 1.3 | -3.5 | 4.8 | 0 |
| 2 | alpha tocopherol | -4.1 | -0.4 | -2 | 1.6 | -2.1 |
| 3 | alpha tocopherol | 0.2 | 25.4 | 20.5 | 4.9 | -20.3 |
| 4 | alpha tocopherol | -3.4 | 32.8 | 20 | 12.8 | -23.4 |
| 5 | alpha tocopherol | - | - | - | - | - |

Supplementary Table 14. Assessment of acetylation agent transport in identified PBLL tunnels

| **Tunnel ID** | **Ligand** | **E_Bound_**  **[kcal/mol]** | **E_Max_**  **[kcal/mol]** | **E_Surface_**  **[kcal/mol]** | **E_a_**  **[kcal/mol]** | **ΔEBS**  **[kcal/mol]** |
| --- | --- | --- | --- | --- | --- | --- |
| 1 | vinyl acetate | -2.8 | -2.6 | -2.6 | 0.0 | -0.2 |
|  | vinyl hydrogen succinate | -4.0 | -3.7 | -3.7 | 0.0 | -0.3 |
|  | vinyl ferulate | -5.5 | -5.1 | -5.2 | 0.1 | -0.3 |
|  | vinyl nicotinate | -4.8 | -4.2 | -4.2 | 0.0 | -0.6 |
| 2 | vinyl acetate | -3.1 | -1.5 | -2.1 | 0.6 | -1.0 |
|  | vinyl hydrogen succinate | -4.4 | -2.0 | -2.3 | 0.3 | -2.1 |
|  | vinyl ferulate | -6.3 | -3.1 | -3.4 | 0.3 | -2.9 |
|  | vinyl nicotinate | -5.0 | -2.0 | -2.4 | 0.4 | -2.6 |
| 3 | vinyl acetate | -2.8 | 8.2 | 0.8 | 7.4 | -3.6 |
|  | vinyl hydrogen succinate | -3.7 | 8.9 | 1.7 | 7.2 | -5.4 |
|  | vinyl ferulate | -3.5 | 15.4 | 9.4 | 6.0 | -12.9 |
|  | vinyl nicotinate | -3.1 | 16.8 | 11.0 | 5.8 | -14.1 |
| 4 | vinyl acetate | -3.1 | 5.5 | -0.4 | 5.9 | -2.7 |
|  | vinyl hydrogen succinate | -4.3 | 7.2 | -0.8 | 8.0 | -3.5 |
|  | vinyl ferulate | -6.2 | 21.0 | 6.4 | 14.6 | -12.6 |
|  | vinyl nicotinate | -5.0 | 14.0 | 3.9 | 10.1 | -8.9 |
| 5 | vinyl acetate | -3.1 | 11.4 | 3.5 | 7.9 | -6.6 |
|  | vinyl hydrogen succinate | -4.4 | 15.1 | 2.2 | 12.9 | -6.6 |
|  | vinyl ferulate | -6.4 | 32.9 | 16.8 | 16.1 | -23.2 |
|  | vinyl nicotinate | -4.9 | 25.5 | 14.0 | 11.5 | 18.9 |

Supplementary Table 15. Assessment of product transport in PBLL identified tunnels

| **Tunnel ID** | **Ligand** | **E_Bound_**  **[kcal/mol]** | **E_Max_**  **[kcal/mol]** | **E_Surface_**  **[kcal/mol]** | **E_a_**  **[kcal/mol]** | **ΔEBS**  **[kcal/mol]** |
| --- | --- | --- | --- | --- | --- | --- |
| 1 | alpha-Tocopherol acetate | -4 | -2.3 | -3.8 | 1.7 | -0.2 |
|  | alpha-Tocopherol succinate | -5.3 | -0.8 | -0.8 | 4.5 | -4.5 |
|  | alpha-Tocopherol ferrulate | -3.6 | -0.4 | -2.1 | 3.2 | -1.5 |
|  | alpha-Tocopherol nicotinate | -6.1 | -0.3 | -0.3 | 5.8 | -5.8 |
| 2 | alpha-Tocopherol acetate | -4 | 1.7 | -3.1 | 5.7 | -0.9 |
|  | alpha-Tocopherol succinate | -3.8 | 3.3 | -2.7 | 7.1 | -1.1 |
|  | alpha-Tocopherol ferrulate | -2.5 | 3.5 | -3.5 | 6 | 1 |
|  | alpha-Tocopherol nicotinate | -3.9 | 3.7 | -2.2 | 7.6 | -1.7 |
| 3 | alpha-Tocopherol acetate | -4.2 | 18.7 | -2.9 | 22.9 | -1.3 |
|  | alpha-Tocopherol succinate | -4.4 | 16.9 | -2.7 | 21.3 | -1.7 |
|  | alpha-Tocopherol ferrulate | -3.4 | 21.2 | -2.7 | 24.6 | -0.7 |
|  | alpha-Tocopherol nicotinate | -5.2 | 18.7 | -2.3 | 23.9 | -2.9 |
| 4 | alpha-Tocopherol acetate | -4.2 | 30 | -3.6 | 34.2 | -0.6 |
|  | alpha-Tocopherol succinate | -4 | 29.9 | -2 | 33.9 | -2 |
|  | alpha-Tocopherol ferrulate | -3.6 | 31 | -3.4 | 34.6 | -0.2 |
|  | alpha-Tocopherol nicotinate | -4.2 | 33.5 | -2.4 | 37.7 | -1.8 |
| 5 | alpha-Tocopherol acetate | - | - | - | - | - |
|  | alpha-Tocopherol succinate | - | - | - | - | - |
|  | alpha-Tocopherol ferrulate | - | - | - | - | - |
|  | alpha-Tocopherol nicotinate | - | - | - | - | - |

Supplementary Table 16. Assessment of alpha tocopherol transport in identified CRL tunnels

| **Tunnel ID** | **Ligand** | **E_Bound_**  **[kcal/mol]** | **E_Max_**  **[kcal/mol]** | **E_Surface_**  **[kcal/mol]** | **E_a_**  **[kcal/mol]** | **ΔEBS**  **[kcal/mol]** |
| --- | --- | --- | --- | --- | --- | --- |
| 1 | alpha tocopherol | -6.1 | -3.8 | -6.2 | 2.4 | 0.1 |
| 2 | alpha tocopherol | - | - | - | - | - |
| 3 | alpha tocopherol | -3.1 | 15.2 | 1.8 | 13.4 | -4.9 |
| 4 | alpha tocopherol | -2.7 | 14.2 | 5.6 | 8.6 | -8.3 |
| 5 | alpha tocopherol | -2.6 | 26.2 | 8.4 | 17.8 | -11 |
| 6 | alpha tocopherol | - | - | - | - | - |
| 7 | alpha tocopherol | - | - | - | - | - |
| 8 | alpha tocopherol | -3.0 | 41.8 | 25.8 | 16.0 | -28.8 |

Supplementary Table 17. Assessment of transport of acetylating agents in identified CRL tunnels

| **Tunnel ID** | **Ligand** | **E_Bound_**  **[kcal/mol]** | **E_Max_**  **[kcal/mol]** | **E_Surface_**  **[kcal/mol]** | **E_a_**  **[kcal/mol]** | **ΔEBS**  **[kcal/mol]** |
| --- | --- | --- | --- | --- | --- | --- |
| 1 | vinyl acetate | -2.5 | -2.5 | -2.8 | 0.3 | 0.3 |
|  | vinyl hydrogen succinate | -4.0 | -3.8 | -4.0 | 0.2 | 0.0 |
|  | vinyl ferulate | -5.0 | -4.5 | -5.2 | 0.7 | 0.2 |
|  | vinyl nicotinate | -3.9 | -3.9 | -4.4 | 0.5 | 0.5 |
| 2 | vinyl acetate | - | - | - | - | - |
|  | vinyl hydrogen succinate | - | - | - | - | - |
|  | vinyl ferulate | - | - | - | - | - |
|  | vinyl nicotinate | - | - | - | - | - |
| 3 | vinyl acetate | -3.0 | 3.1 | -0.8 | 3.9 | -2.2 |
|  | vinyl hydrogen succinate | -4.0 | 2.4 | -0.7 | 3.1 | -3.3 |
|  | vinyl ferulate | -6.1 | 7.7 | 1.8 | 5.9 | -7.9 |
|  | vinyl nicotinate | -4.8 | 7.1 | 2.7 | 4.4 | -7.5 |
| 4 | vinyl acetate | -3.0 | 0.7 | -2.6 | 3.3 | -0.4 |
|  | vinyl hydrogen succinate | -4.3 | 0.1 | -2.8 | 2.9 | -1.5 |
|  | vinyl ferulate | -6.1 | 4.1 | -0.8 | 4.9 | -5.3 |
|  | vinyl nicotinate | -5.0 | 4.1 | -3.0 | 7.1 | -2.0 |
| 5 | vinyl acetate | -3.1 | 12.3 | -1.0 | 13.3 | -2.1 |
|  | vinyl hydrogen succinate | -4.3 | 10.9 | -0.8 | 11.7 | -3.5 |
|  | vinyl ferulate | -6.1 | 15.2 | 3.5 | 11.7 | -9.6 |
|  | vinyl nicotinate | -5.0 | 19.2 | 4.3 | 14.9 | -9.3 |
| 6 | vinyl acetate | -3.1 | 14.9 | 2.2 | 12.7 | -5.3 |
|  | vinyl hydrogen succinate | -4.4 | 16.3 | 1.3 | 15 | -5.7 |
|  | vinyl ferulate | -6.1 | 29.0 | 7.3 | 21.7 | -13.4 |
|  | vinyl nicotinate | -5.0 | 29.8 | 5.9 | 23.9 | -10.9 |
| 7 | vinyl acetate | -3.1 | 14.5 | 5.4 | 9.1 | -8.5 |
|  | vinyl hydrogen succinate | -4.4 | 18.4 | 4.3 | 14.1 | -8.7 |
|  | vinyl ferulate | -6.0 | 37.9 | 18.6 | 19.3 | -24.6 |
|  | vinyl nicotinate | -5.0 | 29.2 | 14.9 | 14.3 | -19.9 |
| 8 | vinyl acetate | -3.1 | 11.3 | 0.3 | 11 | -3.4 |
|  | vinyl hydrogen succinate | -4.4 | 15.4 | -0.7 | 16.1 | -3.7 |
|  | vinyl ferulate | -6.0 | 31.9 | 18.3 | 13.6 | -24.3 |
|  | vinyl nicotinate | -4.9 | 24.5 | 12 | 12.5 | -16.9 |

Supplementary Table 18. Assessment of product transport in identified CRL tunnels

| **Tunnel ID** | **Ligand** | **E_Bound_**  **[kcal/mol]** | **E_Max_**  **[kcal/mol]** | **E_Surface_**  **[kcal/mol]** | **E_a_**  **[kcal/mol]** | **ΔEBS**  **[kcal/mol]** |
| --- | --- | --- | --- | --- | --- | --- |
| 1 | alpha-Tocopherol acetate | -6.8 | -4.7 | -4.9 | 2.1 | -1.9 |
|  | alpha-Tocopherol succinate | -5.7 | -4.6 | -4.6 | 1.1 | -1.1 |
|  | alpha-Tocopherol ferrulate | -7.3 | -5.0 | -5.3 | 2.3 | -2.0 |
|  | alpha-Tocopherol nicotinate | -6.3 | -4.6 | -4.7 | 1.7 | -1.6 |
| 2 | alpha-Tocopherol acetate | - | - | - | - | - |
|  | alpha-Tocopherol succinate | - | - | - | - | - |
|  | alpha-Tocopherol ferrulate | - | - | - | - | - |
|  | alpha-Tocopherol nicotinate | - | - | - | - | - |
| 3 | alpha-Tocopherol acetate | -4.8 | 7.3 | -4.5 | 12.1 | -0.3 |
|  | alpha-Tocopherol succinate | -5.1 | 8.3 | -4.5 | 13.4 | -0.6 |
|  | alpha-Tocopherol ferrulate | -5.7 | 9.6 | -2.6 | 15.3 | -3.1 |
|  | alpha-Tocopherol nicotinate | -5.7 | 8.9 | -5.2 | 14.6 | -0.5 |
| 4 | alpha-Tocopherol acetate | -3.4 | 16.1 | -5.7 | 19.5 | 2.3 |
|  | alpha-Tocopherol succinate | -3.6 | 16.6 | -4.3 | 20.2 | 0.7 |
|  | alpha-Tocopherol ferrulate | -2.5 | 19.1 | -2.4 | 21.6 | -0.1 |
|  | alpha-Tocopherol nicotinate | -4.0 | 19.3 | -4.7 | 23.3 | 0.7 |
| 5 | alpha-Tocopherol acetate | -4.4 | 21.6 | -5.4 | 26.0 | 1.0 |
|  | alpha-Tocopherol succinate | -1.9 | 21.8 | -3.7 | 23.7 | 1.8 |
|  | alpha-Tocopherol ferrulate | -3.5 | 30.4 | -2.9 | 33.9 | -0.6 |
|  | alpha-Tocopherol nicotinate | -3.1 | 33.6 | -5.0 | 36.7 | 1.9 |
| 6 | alpha-Tocopherol acetate | - | - | - | - | - |
|  | alpha-Tocopherol succinate | - | - | - | - | - |
|  | alpha-Tocopherol ferrulate | - | - | - | - | - |
|  | alpha-Tocopherol nicotinate | - | - | - | - | - |
| 7 | alpha-Tocopherol acetate | - | - | - | - | - |
|  | alpha-Tocopherol succinate | - | - | - | - | - |
|  | alpha-Tocopherol ferrulate | - | - | - | - | - |
|  | alpha-Tocopherol nicotinate | - | - | - | - | - |
| 8 | alpha-Tocopherol acetate | -3.3 | 41.5 | -5.1 | 44.8 | 1.8 |
|  | alpha-Tocopherol succinate | - | - | - | - | - |
|  | alpha-Tocopherol ferrulate | - | - | - | - | - |
|  | alpha-Tocopherol nicotinate | - | - | - | - | - |

Supplementary Table 19. Assessment of alpha tocopherol transport in identified RML tunnels

| **Tunnel ID** | **Ligand** | **E_Bound_**  **[kcal/mol]** | **E_Max_**  **[kcal/mol]** | **E_Surface_**  **[kcal/mol]** | **E_a_**  **[kcal/mol]** | **ΔEBS**  **[kcal/mol]** |
| --- | --- | --- | --- | --- | --- | --- |
| 1 | alpha tocopherol | -4.9 | -4.5 | -4.7 | 0.2 | -0.2 |
| 2 | alpha tocopherol | -5.4 | -4.3 | -4.8 | 0.5 | -0.6 |
| 3 | alpha tocopherol | -5.0 | -4.0 | -4.3 | 0.3 | -0.7 |

Supplementary Table 20. Assessment of acetylation agent transport in identified RML tunnels

| **Tunnel ID** | **Ligand** | **E_Bound_**  **[kcal/mol]** | **E_Max_**  **[kcal/mol]** | **E_Surface_**  **[kcal/mol]** | **E_a_**  **[kcal/mol]** | **ΔEBS**  **[kcal/mol]** |
| --- | --- | --- | --- | --- | --- | --- |
| 1 | vinyl acetate | -2.2 | -2.2 | -2.2 | 0.0 | 0.0 |
|  | vinyl hydrogen succinate | -3.0 | -2.9 | -3.0 | -0.1 | 0.0 |
|  | vinyl ferulate | -4.4 | -4.1 | -4.4 | 0.3 | 0.0 |
|  | vinyl nicotinate | -3.4 | -3.4 | -3.5 | 0.1 | 0.1 |
| 2 | vinyl acetate | -2.4 | -2.0 | -2.0 | 0.0 | -0.4 |
|  | vinyl hydrogen succinate | -3.1 | -2.9 | -3.1 | 0.2 | 0.0 |
|  | vinyl ferulate | -4.5 | -4.0 | -4.0 | 0.0 | -0.5 |
|  | vinyl nicotinate | -3.7 | -3.1 | -3.1 | 0.0 | -0.6 |
| 3 | vinyl acetate | -2.3 | -1.8 | -1.9 | 0.1 | -0.4 |
|  | vinyl hydrogen succinate | -3.0 | -2.8 | -2.9 | 0.1 | -0.1 |
|  | vinyl ferulate | -4.5 | -3.3 | -3.5 | 0.2 | -1.0 |
|  | vinyl nicotinate | -3.8 | -2.5 | -2.5 | 0.0 | -1.3 |

Supplementary Table 21. Evaluation of the transport of products in the tunnels identified by the RML

| **Tunnel ID** | **Ligand** | **E_Bound_**  **[kcal/mol]** | **E_Max_**  **[kcal/mol]** | **E_Surface_**  **[kcal/mol]** | **E_a_**  **[kcal/mol]** | **ΔEBS**  **[kcal/mol]** |
| --- | --- | --- | --- | --- | --- | --- |
| 1 | alpha-Tocopherol acetate | -4.2 | -4.2 | -4.6 | 0.0 | 0.4 |
|  | alpha-Tocopherol succinate | -4.8 | -4.3 | -5.1 | 0.5 | 0.3 |
|  | alpha-Tocopherol ferrulate | -4.5 | -4.2 | -4.7 | 0.3 | 0.2 |
|  | alpha-Tocopherol nicotinate | -4.8 | -4.0 | -5.2 | 0.8 | 0.4 |
| 2 | alpha-Tocopherol acetate | -3.8 | -3.8 | -5.0 | 0.0 | 1.2 |
|  | alpha-Tocopherol succinate | -5.0 | -4.1 | -4.9 | 0.9 | -0.1 |
|  | alpha-Tocopherol ferrulate | -5.0 | -4.4 | -5.2 | 0.6 | 0.2 |
|  | alpha-Tocopherol nicotinate | -4.8 | -4.4 | -4.9 | 0.4 | 0.1 |
| 3 | alpha-Tocopherol acetate | -2.5 | -2.5 | -4.5 | 0.0 | 2.0 |
|  | alpha-Tocopherol succinate | -0.4 | -0.4 | -5.0 | 0.0 | 4.6 |
|  | alpha-Tocopherol ferrulate | 3.0 | 3.0 | -5.3 | 0.0 | 8.3 |
|  | alpha-Tocopherol nicotinate | -1.7 | -1.4 | -5.3 | 0.3 | 3.6 |

Supplementary Table 22. Assessment of alpha tocopherol transport in CALB identified tunnels

| **Tunnel ID** | **Ligand** | **E_Bound_**  **[kcal/mol]** | **E_Max_**  **[kcal/mol]** | **E_Surface_**  **[kcal/mol]** | **E_a_**  **[kcal/mol]** | **ΔEBS**  **[kcal/mol]** |
| --- | --- | --- | --- | --- | --- | --- |
| 1 | alpha tocopherol | -4.0 | -1.9 | -4.9 | 3.0 | 0.9 |
| 2 | alpha tocopherol | -6.2 | -4.2 | -5.8 | 1.6 | -0.4 |
| 3 | alpha tocopherol | -6.1 | -4.6 | -5.7 | 1.1 | -0.4 |
| 4 | alpha tocopherol | -0.5 | 0.9 | -3.1 | 4.0 | 2.6 |
| 5 | alpha tocopherol | -5.6 | 30.6 | -1.7 | 32.3 | -3.9 |
| 6 | alpha tocopherol | -6.8 | 29.7 | -2.0 | 31.7 | -4.8 |

Supplementary Table 23. Assessment of acetylation agent transport in CALB identified tunnels

| **Tunnel ID** | **Ligand** | **E_Bound_**  **[kcal/mol]** | **E_Max_**  **[kcal/mol]** | **E_Surface_**  **[kcal/mol]** | **E_a_**  **[kcal/mol]** | **ΔEBS**  **[kcal/mol]** |
| --- | --- | --- | --- | --- | --- | --- |
| 1 | vinyl acetate | -2.8 | -2.4 | -2.5 | 0.1 | -0.3 |
|  | vinyl hydrogen succinate | -3.5 | -3.0 | -3.1 | 0.1 | -0.4 |
|  | vinyl ferulate | -5.1 | -3.8 | -4.2 | 0.4 | -0.9 |
|  | vinyl nicotinate | -3.0 | -2.6 | -3.1 | 0.5 | 0.1 |
| 2 | vinyl acetate | -2.8 | -2.6 | -2.6 | 0.0 | -0.2 |
|  | vinyl hydrogen succinate | -3.9 | -3.4 | -3.7 | 0.3 | -0.2 |
|  | vinyl ferulate | -5.9 | -4.4 | -5.6 | 1.2 | -0.3 |
|  | vinyl nicotinate | -4.2 | -3.9 | -4.0 | 0.1 | -0.2 |
| 3 | vinyl acetate | -2.9 | -2.5 | -2.6 | 0.1 | -0.3 |
|  | vinyl hydrogen succinate | -4.0 | -3.5 | -3.7 | 0.2 | -0.3 |
|  | vinyl ferulate | -5.8 | -4.4 | -5.7 | 1.3 | -0.1 |
|  | vinyl nicotinate | -4.0 | -3.8 | -3.9 | 0.1 | -0.1 |
| 4 | vinyl acetate | -2.2 | -2.0 | -3.0 | 1.0 | 0.8 |
|  | vinyl hydrogen succinate | -3.1 | -2.9 | -3.9 | 1.0 | 0.8 |
|  | vinyl ferulate | -4.5 | -3.7 | -5.5 | 1.8 | 1.0 |
|  | vinyl nicotinate | -3.0 | -2.7 | -4.1 | 1.4 | 1.1 |
| 5 | vinyl acetate | -2.8 | 12.4 | -1.1 | 13.5 | -1.7 |
|  | vinyl hydrogen succinate | -3.8 | 14.3 | -0.7 | 15.0 | -3.1 |
|  | vinyl ferulate | -5.7 | 26.0 | 6.6 | 19.4 | -12.3 |
|  | vinyl nicotinate | -4.4 | 23.4 | 4.1 | 19.8 | -8.5 |
| 6 | vinyl acetate | -2.8 | 10.9 | -1.1 | 12.0 | -1.7 |
|  | vinyl hydrogen succinate | -3.8 | 11.1 | -0.7 | 11.8 | -3.1 |
|  | vinyl ferulate | -5.7 | 20.9 | 13.8 | 7.1 | -19.5 |
|  | vinyl nicotinate | -4.2 | 21.2 | 6.2 | 15.0 | -10.4 |

Supplementary Table 24. Evaluation of the transport of products in the tunnels identified by CALB

| **Tunnel ID** | **Ligand** | **E_Bound_**  **[kcal/mol]** | **E_Max_**  **[kcal/mol]** | **E_Surface_**  **[kcal/mol]** | **E_a_**  **[kcal/mol]** | **ΔEBS**  **[kcal/mol]** |
| --- | --- | --- | --- | --- | --- | --- |
| 1 | alpha-Tocopherol acetate | -6.8 | -2.9 | -4.9 | 3.9 | -1.9 |
|  | alpha-Tocopherol succinate | -6.1 | -3.7 | -5.2 | 2.4 | -0.9 |
|  | alpha-Tocopherol ferrulate | -6.0 | -3.0 | -4.0 | 3.0 | -2.0 |
|  | alpha-Tocopherol nicotinate | -7.0 | -4.3 | -5.5 | 2.7 | -1.5 |
| 2 | alpha-Tocopherol acetate | -5.7 | -3.7 | -5.9 | 2.0 | 0.2 |
|  | alpha-Tocopherol succinate | -6.2 | -4.4 | -4.7 | 1.8 | -1.5 |
|  | alpha-Tocopherol ferrulate | -6.6 | -4.7 | -5.2 | 1.9 | -1.4 |
|  | alpha-Tocopherol nicotinate | -6.6 | -4.7 | -5.2 | 1.9 | -1.4 |
| 3 | alpha-Tocopherol acetate | -6.9 | -4.2 | -5.7 | 2.7 | -1.2 |
|  | alpha-Tocopherol succinate | -6.6 | -4.8 | -4.7 | 1.8 | -1.9 |
|  | alpha-Tocopherol ferrulate | -5.7 | -3.5 | -5.4 | 2.2 | -0.3 |
|  | alpha-Tocopherol nicotinate | -5.8 | -4.5 | -4.7 | 1.3 | -1.1 |
| 4 | alpha-Tocopherol acetate* | -5.5 | 0.1 | -6.3 | 5.6 | 0.8 |
|  | alpha-Tocopherol succinate* | -5.5 | -1.1 | -2.7 | 4.4 | -2.8 |
|  | alpha-Tocopherol ferrulate | -6.0 | 1.9 | -2.9 | 7.9 | -3.1 |
|  | alpha-Tocopherol nicotinate | -5.9 | 0.3 | -5.1 | 6.2 | -0.8 |
| 5 | alpha-Tocopherol acetate* | -4.9 | 27.4 | -4.8 | 32.3 | -0.1 |
|  | alpha-Tocopherol succinate* | -5.7 | 27.9 | -5.4 | 33.6 | -0.3 |
|  | alpha-Tocopherol ferrulate | -4.5 | 43.3 | -5.6 | 38.8 | 1.1 |
|  | alpha-Tocopherol nicotinate | -5.1 | 31.3 | -5.8 | 36.4 | 0.7 |
| 6 | alpha-Tocopherol acetate* | -3.4 | 28.7 | -5.4 | 32.1 | 2.0 |
|  | alpha-Tocopherol succinate* | -1.6 | 29.9 | -5.2 | 31.5 | 3.6 |
|  | alpha-Tocopherol ferrulate | -0.5 | 39.1 | -4.4 | 39.6 | 3.9 |
|  | alpha-Tocopherol nicotinate | -1.3 | 33.2 | -5.8 | 34.5 | 4.5 |

Supplementary Table 25. Assessment of alpha tocopherol transport in identified TLL tunnels

| **Tunnel ID** | **Ligand** | **E_Bound_**  **[kcal/mol]** | **E_Max_**  **[kcal/mol]** | **E_Surface_**  **[kcal/mol]** | **E_a_**  **[kcal/mol]** | **ΔEBS**  **[kcal/mol]** |
| --- | --- | --- | --- | --- | --- | --- |
| 1 | alpha tocopherol | -5.1 | -4.7 | -4.7 | 0.0 | -0.4 |
| 2 | alpha tocopherol | -5.4 | -4.5 | -4.8 | 0.3 | -0.6 |
| 3 | alpha tocopherol | -5.0 | -2.7 | -4.8 | 2.1 | -0.2 |
| 4 | alpha tocopherol | - | - | - | - | - |
| 5 | alpha tocopherol | - | - | - | - | - |
| 6 | alpha tocopherol | - | - | - | - | - |
| 7 | alpha tocopherol | - | - | - | - | - |
| 8 | alpha tocopherol | - | - | - | - | - |

Supplementary Table 26. Assessment of acetylation agent transport in identified TLL tunnels

| **Tunnel ID** | **Ligand** | **E_Bound_**  **[kcal/mol]** | **E_Max_**  **[kcal/mol]** | **E_Surface_**  **[kcal/mol]** | **E_a_**  **[kcal/mol]** | **ΔEBS**  **[kcal/mol]** |
| --- | --- | --- | --- | --- | --- | --- |
| 1 | vinyl acetate | -1.7 | -1.7 | -1.8 | 0.1 | 0.1 |
|  | vinyl hydrogen succinate | -2.7 | -2.7 | -2.7 | 0.0 | 0.0 |
|  | vinyl ferulate | -3.8 | -3.6 | -3.6 | 0.0 | -0.2 |
|  | vinyl nicotinate | -2.7 | -2.7 | -2.7 | 0.0 | 0.0 |
| 2 | vinyl acetate | -2.7 | -2.4 | -2.4 | 0.0 | -0.3 |
|  | vinyl hydrogen succinate | -3.4 | -3.3 | -3.3 | 0.0 | -0.1 |
|  | vinyl ferulate | -4.8 | -4.4 | -4.4 | 0.0 | -0.4 |
|  | vinyl nicotinate | -4.1 | -3.5 | -3.7 | 0.2 | -0.4 |
| 3 | vinyl acetate | -2.6 | -1.9 | -1.9 | 0.0 | -0.7 |
|  | vinyl hydrogen succinate | -3.5 | -2.8 | -3.0 | 0.2 | -0.5 |
|  | vinyl ferulate | -4.9 | -3.1 | -3.8 | 0.7 | -1.1 |
|  | vinyl nicotinate | -4.2 | -2.6 | -3 | 0.4 | -1.2 |
| 4 | vinyl acetate | -2.2 | 12.9 | -1.5 | 14.4 | -0.7 |
|  | vinyl hydrogen succinate | -3.1 | 15.3 | -2 | 17.3 | -1.1 |
|  | vinyl ferulate | -4.4 | 35 | 3.2 | 31.8 | -7.6 |
|  | vinyl nicotinate | -3.7 | 25.6 | 1.2 | 24.4 | -4.9 |
| 5 | vinyl acetate | -2.7 | 15.2 | -2.3 | 17.5 | -0.4 |
|  | vinyl hydrogen succinate | -3.8 | 17.6 | -1.1 | 18.7 | -2.7 |
|  | vinyl ferulate | -4.9 | 37.3 | 8.4 | 28.9 | -13.3 |
|  | vinyl nicotinate | -4.3 | 30 | 3.3 | 26.7 | -7.6 |
| 6 | vinyl acetate | -2.7 | 15.6 | -2.4 | 18.0 | -0.3 |
|  | vinyl hydrogen succinate | -3.7 | 16.7 | -3.0 | 19.7 | -0.7 |
|  | vinyl ferulate | -4.5 | 38.2 | -2.0 | 40.2 | -2.5 |
|  | vinyl nicotinate | -4.4 | 33.0 | -2.9 | 35.9 | -1.5 |
| 7 | vinyl acetate | -2.6 | 18.1 | -2.6 | 20.7 | 0.0 |
|  | vinyl hydrogen succinate | -3.8 | 17.7 | -3.2 | 20.9 | -0.6 |
|  | vinyl ferulate | -4.7 | 36.7 | -3.9 | 40.6 | -0.8 |
|  | vinyl nicotinate | -4.4 | 29.5 | -3.1 | 32.6 | -1.3 |
| 8 | vinyl acetate | -2.6 | 18.1 | -1.7 | 19.8 | -0.9 |
|  | vinyl hydrogen succinate | -3.7 | 17.6 | -2.8 | 20.4 | -0.9 |
|  | vinyl ferulate | -4.8 | 37.4 | 0.3 | 37.1 | -5.1 |
|  | vinyl nicotinate | -4.3 | 30.1 | -0.9 | 31.0 | -3.4 |

Supplementary Table 27. Assessment of product transport in identified TLL tunnels

| **Tunnel ID** | **Ligand** | **E_Bound_**  **[kcal/mol]** | **E_Max_**  **[kcal/mol]** | **E_Surface_**  **[kcal/mol]** | **E_a_**  **[kcal/mol]** | **ΔEBS**  **[kcal/mol]** |
| --- | --- | --- | --- | --- | --- | --- |
| 1 | alpha-Tocopherol acetate | -4.3 | -4.1 | -4.9 | 0.2 | 0.6 |
|  | alpha-Tocopherol succinate | -4.9 | -4.3 | -5.5 | 0.6 | 0.6 |
|  | alpha-Tocopherol ferrulate | -5.4 | -4.9 | -5.5 | 0.5 | 0.1 |
|  | alpha-Tocopherol nicotinate | -5.1 | -4.9 | -5 | 0.2 | -0.1 |
| 2 | alpha-Tocopherol acetate | -4.8 | -4.1 | -4.1 | 0.7 | -0.7 |
|  | alpha-Tocopherol succinate | -5.1 | -4.4 | -5.4 | 0.7 | 0.3 |
|  | alpha-Tocopherol ferrulate | -5 | -4.5 | -5.1 | 0.5 | 0.1 |
|  | alpha-Tocopherol nicotinate | -5.6 | -4.8 | -5.1 | 0.8 | -0.5 |
| 3 | alpha-Tocopherol acetate | -4.8 | -4.3 | -4.9 | 0.5 | 0.1 |
|  | alpha-Tocopherol succinate | -4.3 | -3.3 | -4.8 | 1 | 0.5 |
|  | alpha-Tocopherol ferrulate | -4.7 | -1.5 | -5.8 | 3.2 | 1.1 |
|  | alpha-Tocopherol nicotinate | -4.9 | -3.6 | -5.2 | 1.3 | 0.3 |
| 4 | alpha-Tocopherol acetate | -3.2 | 37.5 | -5 | 40.7 | 1.8 |
|  | alpha-Tocopherol succinate | - | - | - | - | - |
|  | alpha-Tocopherol ferrulate | - | - | - | - | - |
|  | alpha-Tocopherol nicotinate | - | - | - | - | - |
| 5 | alpha-Tocopherol acetate | - | - | - | - | - |
|  | alpha-Tocopherol succinate | - | - | - | - | - |
|  | alpha-Tocopherol ferrulate | - | - | - | - | - |
|  | alpha-Tocopherol nicotinate | - | - | - | - | - |
| 6 | alpha-Tocopherol acetate | - | - | - | - | - |
|  | alpha-Tocopherol succinate | - | - | - | - | - |
|  | alpha-Tocopherol ferrulate | - | - | - | - | - |
|  | alpha-Tocopherol nicotinate | - | - | - | - | - |
| 7 | alpha-Tocopherol acetate | - | - | - | - | - |
|  | alpha-Tocopherol succinate | - | - | - | - | - |
|  | alpha-Tocopherol ferrulate | - | - | - | - | - |
|  | alpha-Tocopherol nicotinate | - | - | - | - | - |
| 8 | alpha-Tocopherol acetate | - | - | - | - | - |
|  | alpha-Tocopherol succinate | - | - | - | - | - |
|  | alpha-Tocopherol ferrulate | - | - | - | - | - |
|  | alpha-Tocopherol nicotinate | - | - | - | - | - |
